# Supplementary material for: Unique DUOX2+ACE2+ small cholangiocytes are pathogenic targets for primary biliary cholangitis
Source: Nat Commun. 2023 Feb 9;14:29. doi: 10.1038/s41467-022-34606-w (PMC9911648; doi:10.1038/s41467-022-34606-w)
Supplement: Supplementary file 1 — Supplementary Information [file 41467_2022_34606_MOESM1_ESM.pdf]

Unique DUOX2$^+$ACE2$^+$ small cholangiocytes are pathogenic targets for primary biliary cholangitis

Supplementary Figures

Supplementary Fig. 1

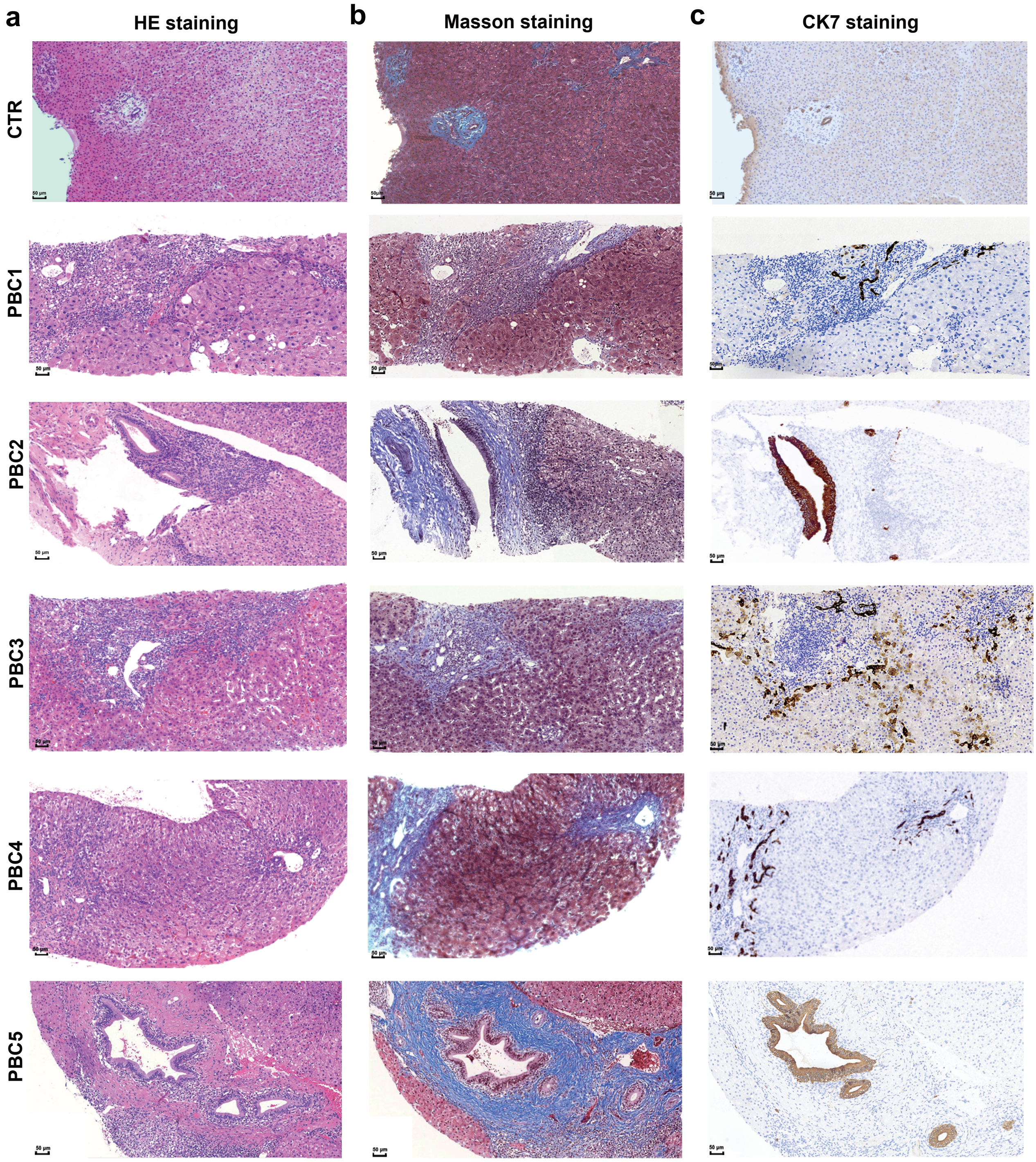

Supplementary Fig. 1: Histological and immunohistochemistry analyses of serial liver sections from the PBC and control patients used in scRNA-seq. a Representative images of hematoxylin and eosin (HE) staining for the liver sections from 5 primary biliary cholangitis (PBC) patients and one of 4 control (CTR) patients. Scale bars: 50 µm. CTR: *n* = 3 independent experiments. **b** Representative images of Masson staining for the liver sections from 5 PBC patients and one of the CTR patients. Scale bars: 50 µm. *n* = 3 independent experiments. **c** Representative immunohistochemistry images of CK7 expression in the liver sections from 5 PBC patients and one of the CTR patients. Scale bars: 50 µm. *n* = 3 independent experiments.

Supplementary Fig. 2

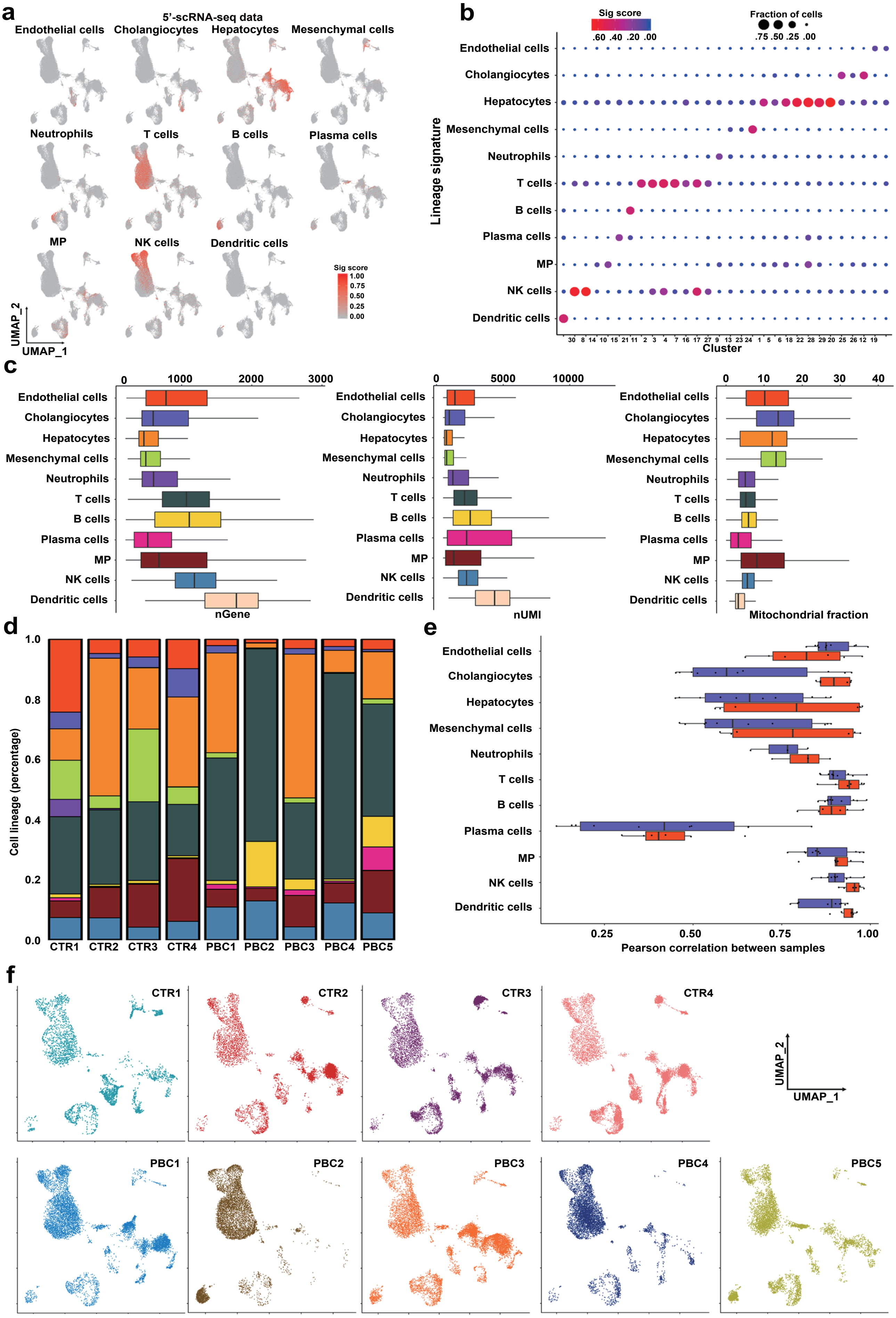

Supplementary Fig. 2: Quality control and annotation of human liver cells in 5'-scRNA-seq. a Gene signature expression of the identified 11 cell lineages covering 70,050 liver cells from 4 human control (CTR) and 5 primary biliary cholangitis (PBC) livers. Red color indicates high expression; gray color indicates low expression. MP, mononuclear phagocytes; NK cells, natural killer cells. **b** Dot plot displayed the distribution of cell lineage marker genes in different liver cell lineages. Circle sizes indicate cell fraction expressing cell lineage marker genes greater than mean; color indicates mean marker gene expression (red, high; blue, low). **c** Box plots exhibited the number of genes (nGene), number of UMIs (nUMI) and mitochondrial gene fraction in the identified 11 cell lineages covering 70,050 liver cells from 4 human control and 5 PBC livers. The center line shows the median, the box limits represent the upper and the lower quartiles, and the whiskers extend to the largest and smallest values. n = 9 human liver samples. **d** The proportions of each cell lineage in each sample. **e** Box plots displaying the agreement of gene expression profiles for different cell lineages across 4 CTR and 5 PBC liver samples. The x axis represents the pearson correlation coefficient of the gene expression level of one cell lineage from one sample relative to that from each other sample among group. The center line shows the median, the box limits represent the upper and the lower quartiles, and the whiskers extend to the largest and smallest values. CTR: n = 4 liver samples from CTR patients, PBC: n = 5 liver samples from PBC patients. **f** UMAP analysis of liver cells in each sample. Source data are provided as a Source Data file.

Supplementary Fig. 3

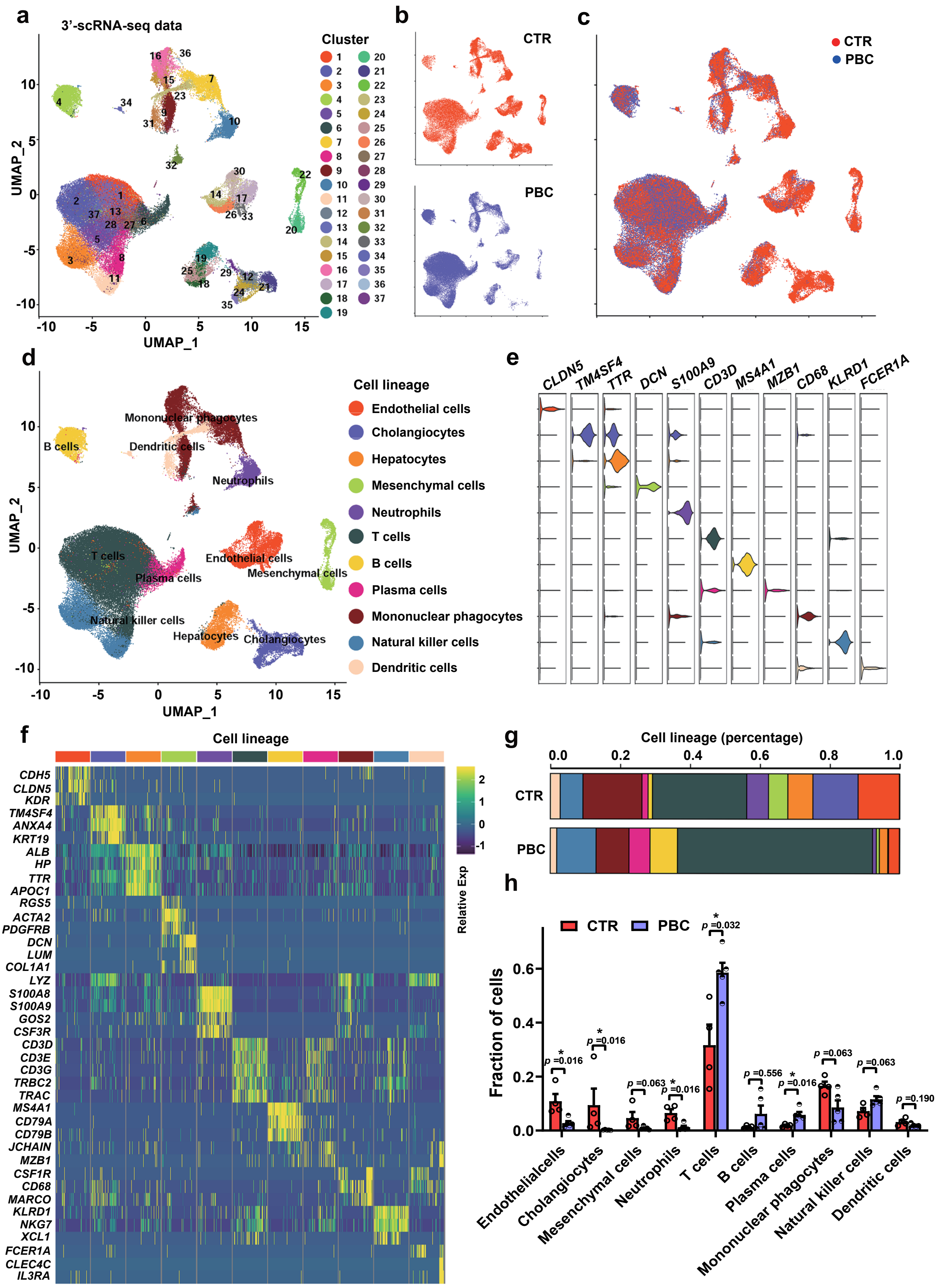

Supplementary Fig. 3: Single-cell transcriptional atlas of human liver cells in 3'-scRNA-seq. **a** UMAP polt of 70,050 liver cells from 4 control (CTR) and 5 untreated primary biliary cholangitis (PBC) patients. **b** Cluster distribution of cells in CTR livers (orange) and PBC livers (blue). **c** UMAP plot displayed cell distribution according to disease condition. **d** UMAP plot exhibited the distribution of identified 11 liver cell lineages. **e** Violin plot displayed the distribution of expression levels of enriched marker genes across 11 cell lineages, colored by cell lineage. **f** Heatmap analysis of the relative expression level of marker genes in 11 cell lineages (top, color-coded by cell lineages), with exemplar genes labelled (left). Columns denote cells; rows denote genes. **g** The relative percentage of different cell lineages for each group, colored by cell lineage. CTR: n = 4 liver samples from CTR patients, PBC: n = 5 liver samples from PBC patients. **h** Comparision of the proportions of different cell lineages between CTR and PBC livers. CTR: n = 4 liver samples from CTR patients, PBC: n = 5 liver samples from PBC patients; Plotted: mean ± S.E.M.; Statistics: two-tailed Mann-Whitney U test, 95% confidence interval; $^{*}p < 0.05$. Source data are provided as a Source Data file.

Supplementary Fig. 4

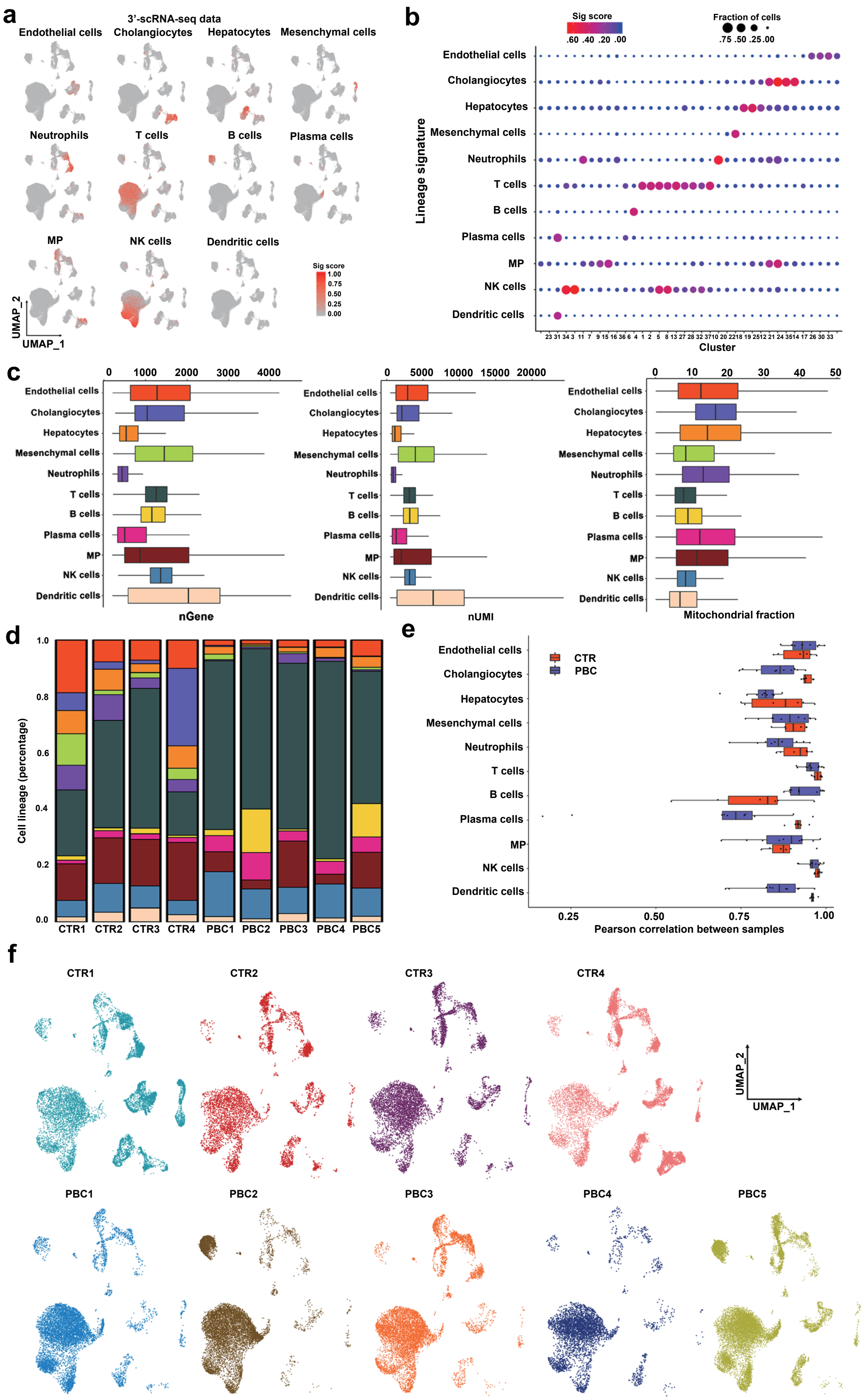

Supplementary Fig. 4: Quality control and annotation of human liver cells in 3'-scRNA-seq. a Gene signature expression of the identified 11 cell lineages covering 70,050 liver cells from 4 human control (CTR) and 5 primary biliary cholangitis (PBC) livers. Red color indicates high expression; gray color indicates low expression. MP, mononuclear phagocytes; NK cells, natural killer cells. **b** Dot plot displayed the distribution of cell lineage marker genes in different liver cell lineages. Circle sizes indicate cell fraction expressing cell lineage marker genes greater than mean; color indicates mean marker gene expression (red, high; blue, low). **c** Box plots exhibited the number of genes (nGene), number of UMIs (nUMI) and mitochondrial gene fraction for the identified 11 cell lineages covering 70,050 liver cells from 4 human control and 5 PBC livers. The center line shows the median, the box limits represent the upper and the lower quartiles, and the whiskers extend to the largest and smallest values. n = 9 human liver samples. **d** The proportions of each cell lineage in each sample. **e** Box plots displaying the agreement in gene expression profiles for different cell lineages across 4 CTR and 5 PBC liver samples. The x axis represents the pearson correlation coefficient of the gene expression level of one cell lineage from one sample relative to that from each other sample among group. The center line shows the median, the box limits represent the upper and the lower quartiles, and the whiskers extend to the largest and smallest values. CTR: n = 4 liver samples from CTR patients, PBC: n = 5 liver samples from PBC patients. **f** UMAP analysis of liver cells in each sample. Source data are provided as a Source Data file.

Supplementary Fig. 5

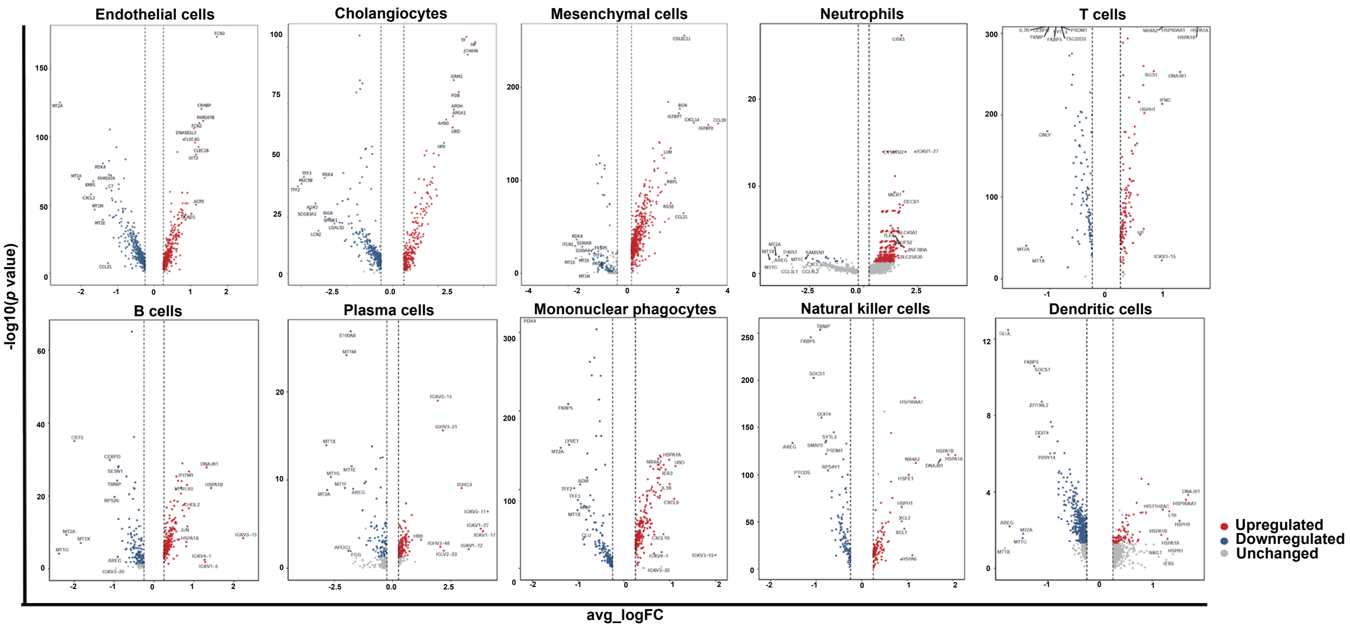

Supplementary Fig. 5: Volcano plots exhibit the up- and downregulated genes for each subtype of liver cells between control and PBC patients in 5'-scRNA-seq. The x axis represents the natural logarithm of fold-change (FC) of the mean expression level of differentially expressed genes (DEGs) between groups (red, upregulated; blue, down-regulated). The y axis represents the significance of the expression change of DEGs. CTR: $n = 4$ liver samples from CTR patients, PBC: $n = 5$ liver samples from PBC patients ; Statistics: two-tailed Wilcox Rank Sum test, 95% confidence interval.

Supplementary Fig. 6

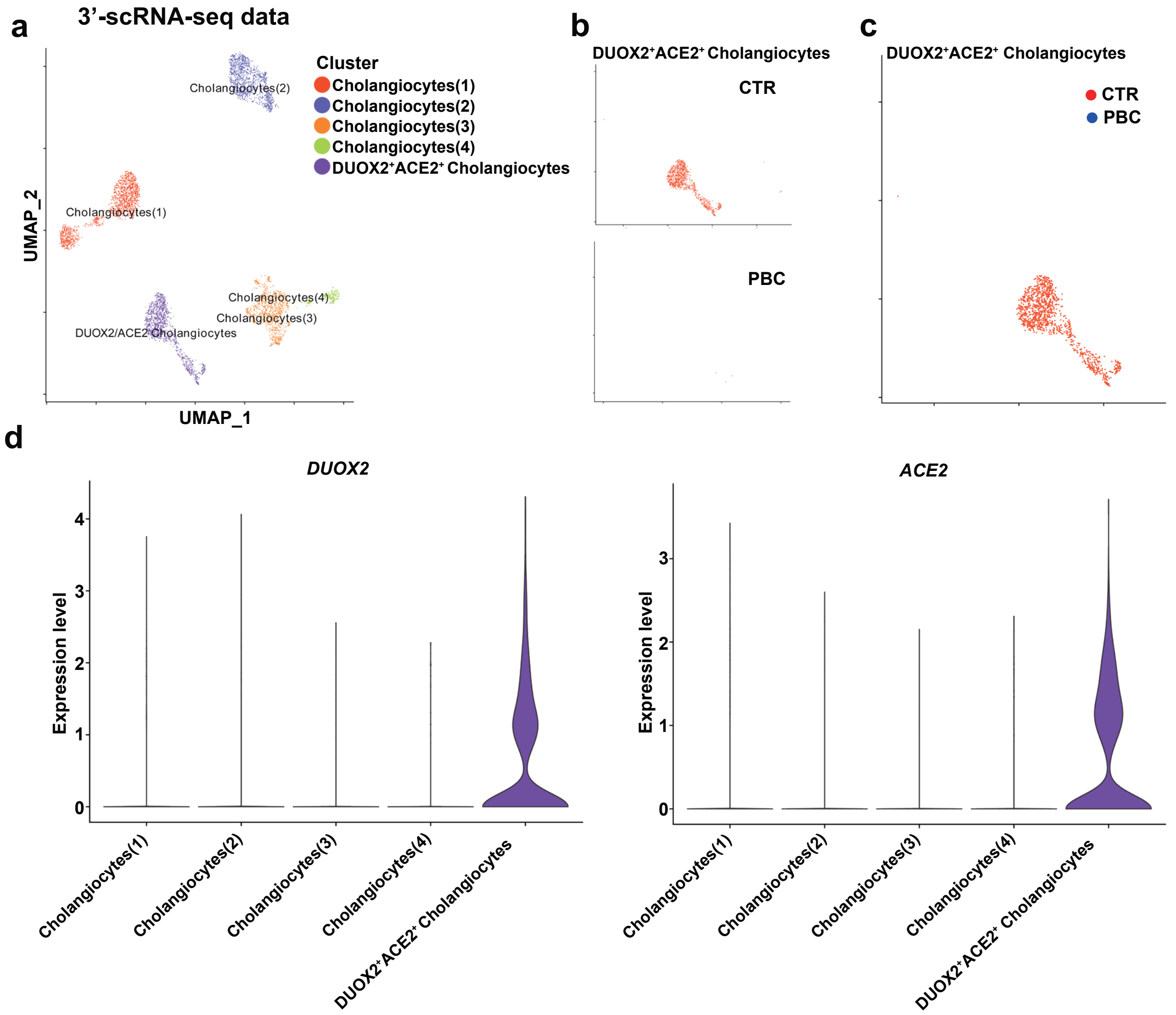

Supplementary Fig. 6: Identification of DUOX2$^+$ACE2$^+$ small cholangiocytes in human control and PBC livers using the 3'-scRNA-seq data. a Clustering of 2,209 cholangiocytes from 4 control (CTR) and 5 primary biliary cholangitis (PBC) livers. **b** The distribution of DUOX2$^+$ACE2$^+$ small cholangiocytes in CTR and PBC livers. **c** UMAP plot displayed the distribution of DUOX2$^+$ACE2$^+$ small cholangiocytes according to disease conditions. **d** Violin plots exhibited the expression levels of DUOX2 and ACE2 in distinct cholangiocyte subpopulations.

Supplementary Fig. 7

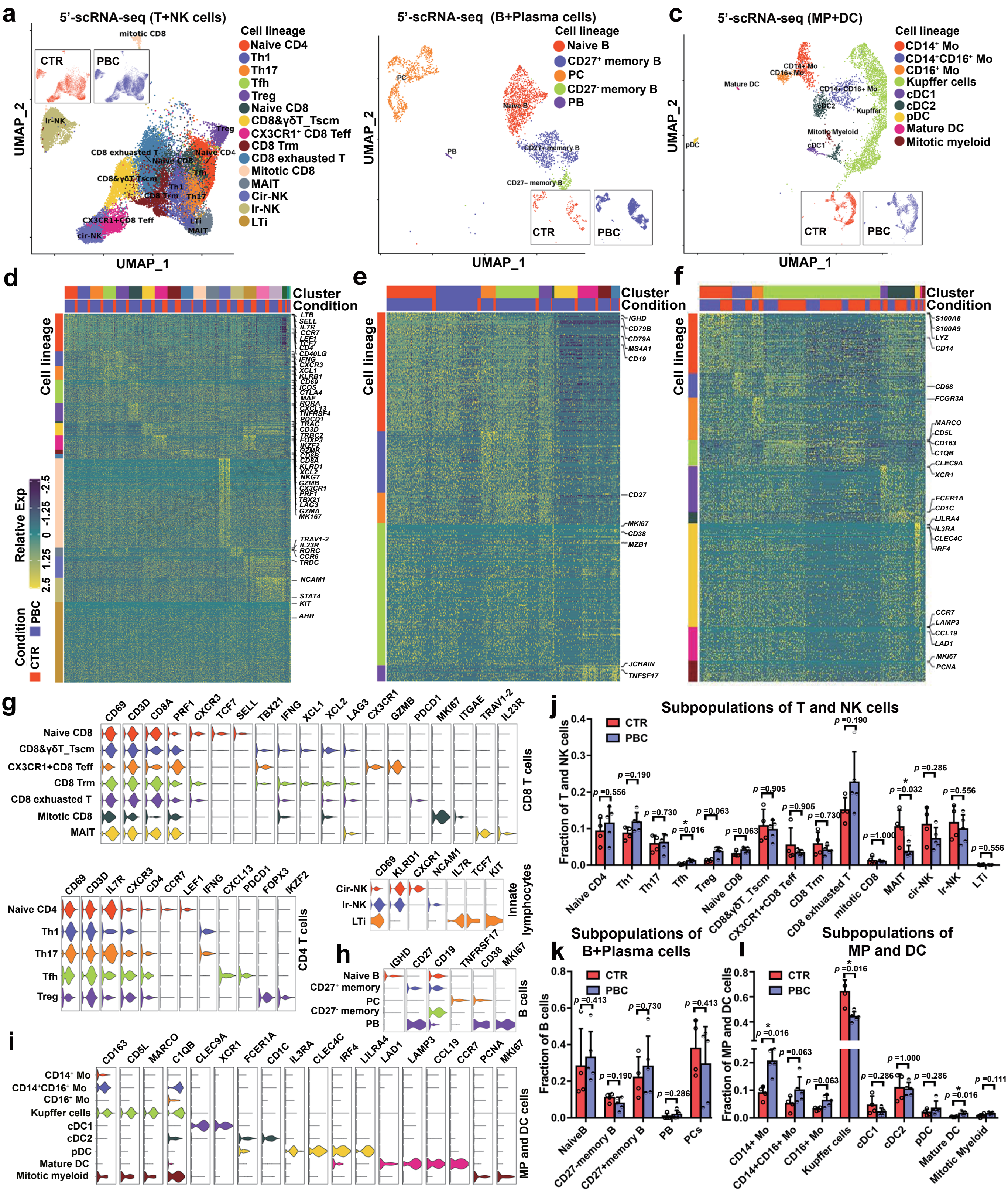

Supplementary Fig. 7: Identification of immune cell subpopulations. a, b, c UMAP analysis of 25,549 T and natural killer (NK) cells (**a**), 2,783 B and plasma cells (**b**), and 4,873 mononuclear phagocytes (MP) and dendritic cells (DC) (**c**) from 4 control (CTR) and 5 primary biliary cholangitis (PBC) livers, with the distribution of annotated cell subpopulations according to disease conditions. Th1, T helper 1 cells; Th17, T helper 17 cells; Tfh, follicular helper T cells; Treg, regulatory T cells; Tscm, T memory stem cells; Teff, effector T cells; Trm, tissue resident memory T cells; MAIT, mucosal-associated invariant T cells; Cir-NK, circulating NK cells; lr-NK, liver-resident NK cells; LTi, lymphoid tissue inducer cells; PB, plasmablasts; PC, plasma cells; Mo, monocytes; cDC1, conventional type 1 dendritic cells; cDC2, conventional type 2 dendritic cells; pDC, plasmacytoid dendritic cells. **d, e, f** Heatmap displayed the relative expression levels of cluster marker genes for T and NK cells (**d**), B and plasma cells (**e**), and MP and DC (**f**) (top, color-coded by cluster and condition), with exemplar genes labelled (right). Columns denote cells; rows denote genes. **g, h, i** Violin plots exhibited the expression levels of enriched marker genes across distinct $CD8^+$ T, $CD4^+$ T cell and innate lymphocyte subpopulations (**g**), B cell and plasma cell subpopulations (**h**), and MP and DC subpopulations (**i**). **j, k, l** The proportions of T and NK cells (**j**), B and plasma cells (**k**), and MP and DC (**l**) subpopulations in CTR and PBC livers. CTR: n = 4 liver samples from CTR patients, PBC: n = 5 liver samples from PBC patients; Plotted: mean ± S.E.M.; Statistics: two-tailed Mann-Whitney U test, 95% confidence interval; $*p < 0.05$. Source data are provided as a Source Data file.

Supplementary Fig. 8

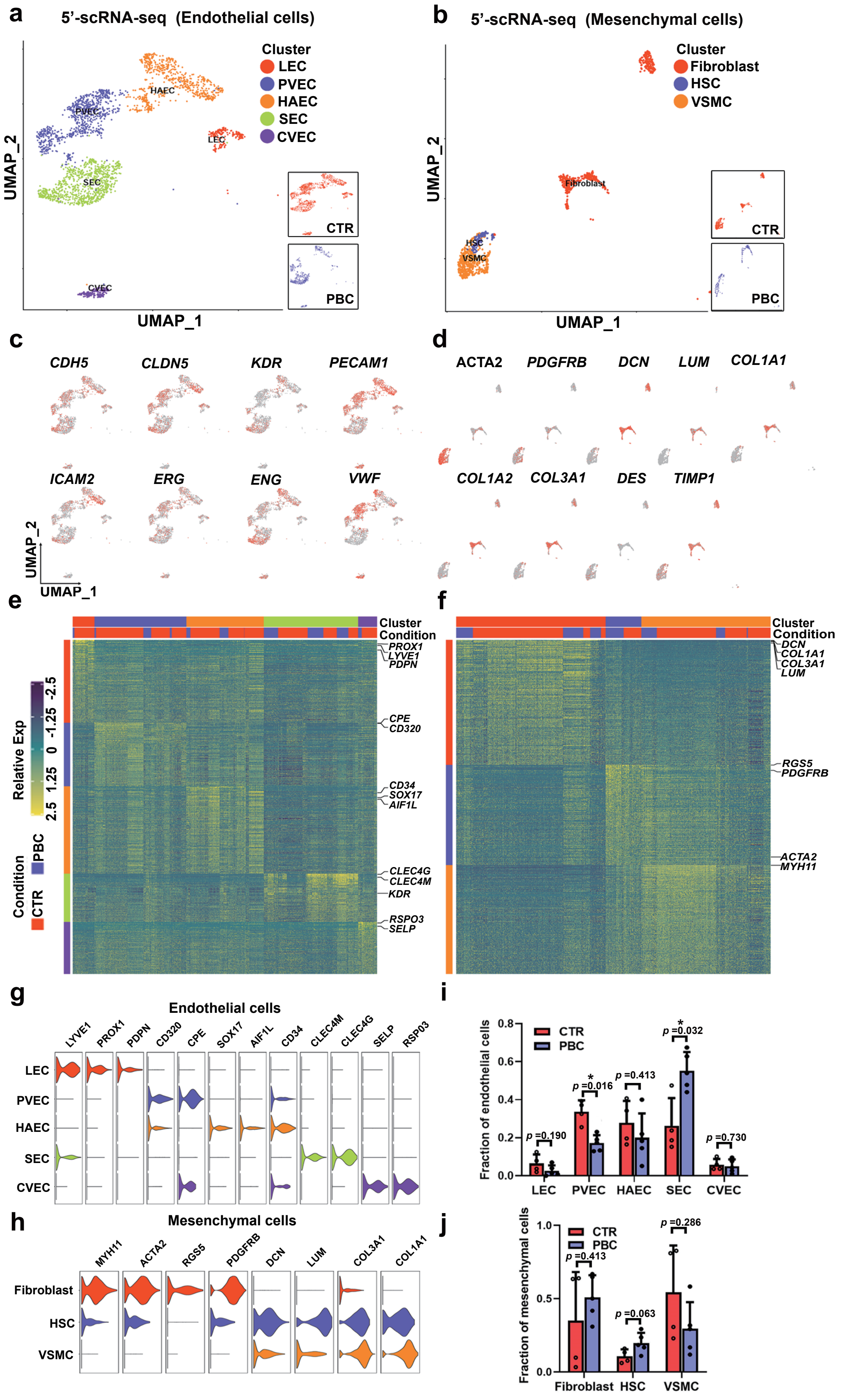

Supplementary Fig. 8: Identification of different subpopulations of endothelial and mesenchymal cells. a, b UMAP analysis of 2,664 endothelial cells (**a**), and 1,136 mesenchymal cells (**b**) from 4 control (CTR) and 5 primary biliary cholangitis (PBC) livers, with the distribution of annotated cell subpopulations according to disease conditions. LEC, lymphatic endothelial cells; PVEC, portal vein endothelial cells; HAEC, hepatic artery endothelial cells; SEC, sinusoidal endothelial cells; CVEC, central vein endothelial cells; HSC, hepatic stellate cells; VSMC, vascular smooth muscle cells. **c, d** Canonical marker genes expressed in different clusters of endothelial cells (**c**), and mesenchymal cells (**d**). **e, f** Heatmap exhibited the relative expression levels of cluster marker genes for endothelial cells (**e**), and mesenchymal cells (**f**) (top, color-coded by cluster and condition), with exemplar genes labelled (right). Columns denote cells; rows denote genes. **g, h** Violin plots displayed the expression levels of enriched marker genes across distinct endothelial cell subpopulations (**g**), and mesenchymal cell subpopulations (**h**). **i, j** The proportions of endothelial cell subpopulations (**i**), and mesenchymal cell subpopulations (**j**) in CTR and PBC livers. CTR: $n = 4$ liver samples from CTR patients, PBC: $n = 5$ liver samples from PBC patients; Plotted: mean ± S.E.M.; Statistics: two-tailed Mann-Whitney *U* test, 95% confidence interval; $*p < 0.05$. Source data are provided as a Source Data file.

Supplementary Fig. 9

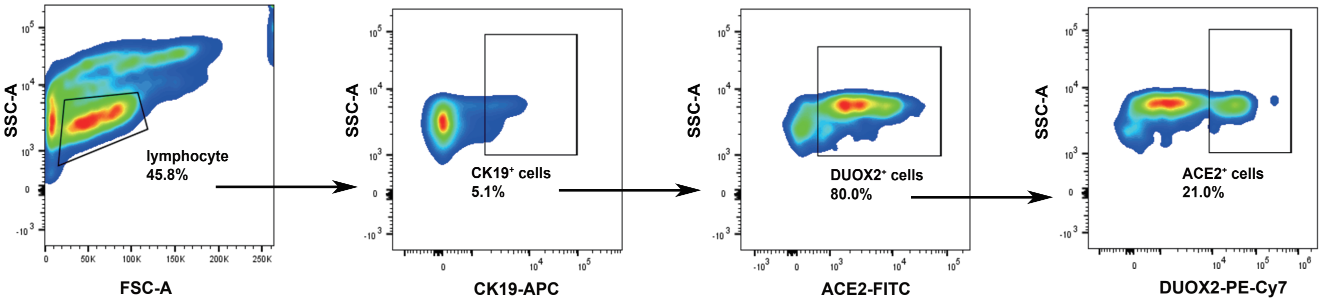

Supplementary Fig. 9: The gating strategies for fluorescence-activated cell sorting used in the Fig. 2g.

Supplementary Fig. 10

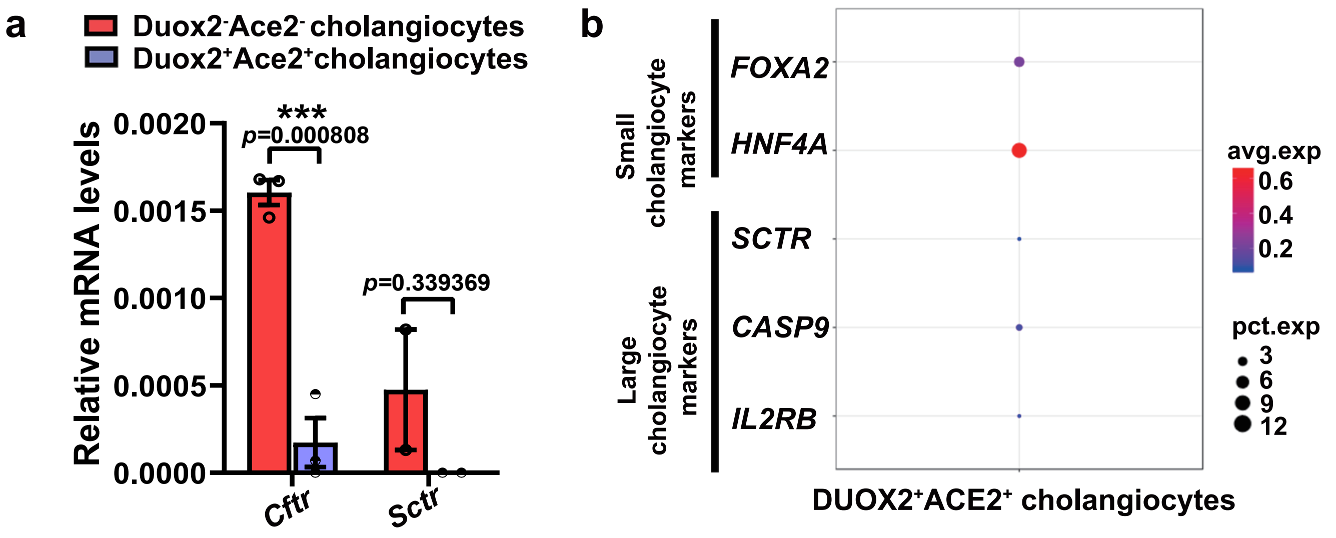

Supplementary Fig. 10: Analyses of canonical marker genes of large and small cholangiocytes in DUOX2$^+$ACE2$^+$ cholangiocytes. a RT-qPCR analysis of canonical marker genes (*Cftr* and *Sctr*) of large cholangiocytes in sorted mouse Duox2$^+$Ace2$^+$ cholangiocytes. *n* = 3 independent experiments; Plotted: mean ± S.E.M.; Statistics: two-tailed independent-sample Student's *t* test, 95% confidence interval; ***p < 0.001. **b** Dot plot displayed the expression levels of marker genes of large and small cholangiocytes in human DUOX2$^+$ACE2$^+$ cholangiocytes using differently expressed genes from 5'-scRNA-seq data. Circle sizes denote expression percentages; colors (red, high; blue, low) denote average expression levels.Source data are provided as a Source Data file.

Supplementary Fig. 11

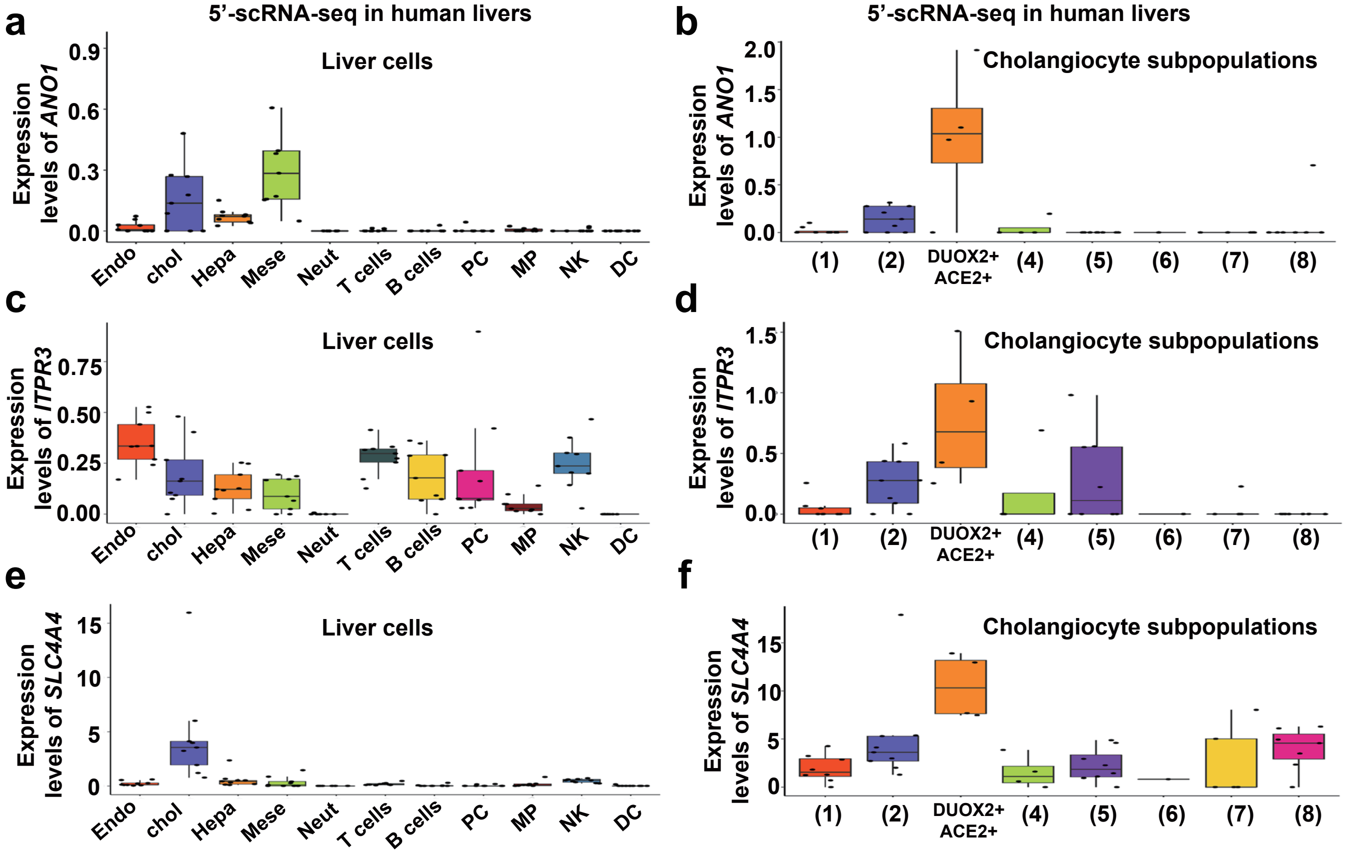

Supplementary Fig. 11: Analyses of key genes regulating bicarbonate and/or chloride secretion into bile in liver cell and cholangiocyte subpopulations using DEGs from 5'-scRNA-seq data. a, b The relative expression levels of *ANO1* in distinct liver cell (**a**) and cholangiocyte subpopulations (**b**) using differently expressed genes (DEGs) from 5'-scRNA-seq data. Each point represents the gene relative expression level of each sample. The center line shows the median of all data points, the box limits represent the upper and the lower quartiles, and the whiskers extend to the largest and smallest values. $n = 9$ human liver samples. Endo, endothelial cells; Chol, cholangiocytes; Hepa, hepatocytes; Mese, mesenchymal cells; Neut, neutrophils; PC, plasma cells; MP, mononuclear phagocytes; NK, natural killer cells; DC, dendritic cells. **c, d** The relative expression levels of *ITPR3* in distinct liver cells (**c**) and cholangiocyte subpopulations (**d**) using DEGs from 5'-scRNA-seq data as in **a** and **b**. The center line shows the median of all data points, the box limits represent the upper and the lower quartiles, and the whiskers extend to the largest and smallest values. *n* =

9 human liver samples. **e, f** The relative expression levels of *SLC4A4* in distinct liver cell (**e**) and cholangiocyte subpopulations (**f**) using DEGs from 5'-scRNA-seq data as in **a** and **b**. The center line shows the median of all data points, the box limits represent the upper and the lower quartiles, and the whiskers extend to the largest and smallest values. n = 9 human liver samples. Source data are provided as a Source Data file.

Supplementary Fig. 12

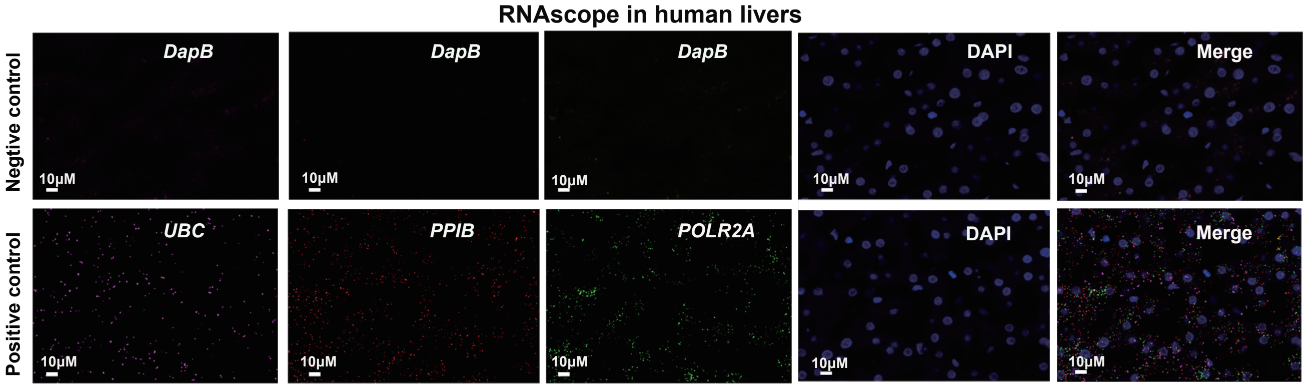

Supplementary Fig.12: Negative and positive controls for RNAscope in the Fig. 3b.

The human healthy liver tissue sections for RNAscope were stained with probes for *DapB* as a negative control, and for *UBC* (purple), *PPIB* (red) and *POLR2A* (green) as positive controls. Scale bars: 10 μm. *n* = 3 independent experiments.

Supplementary Fig. 13

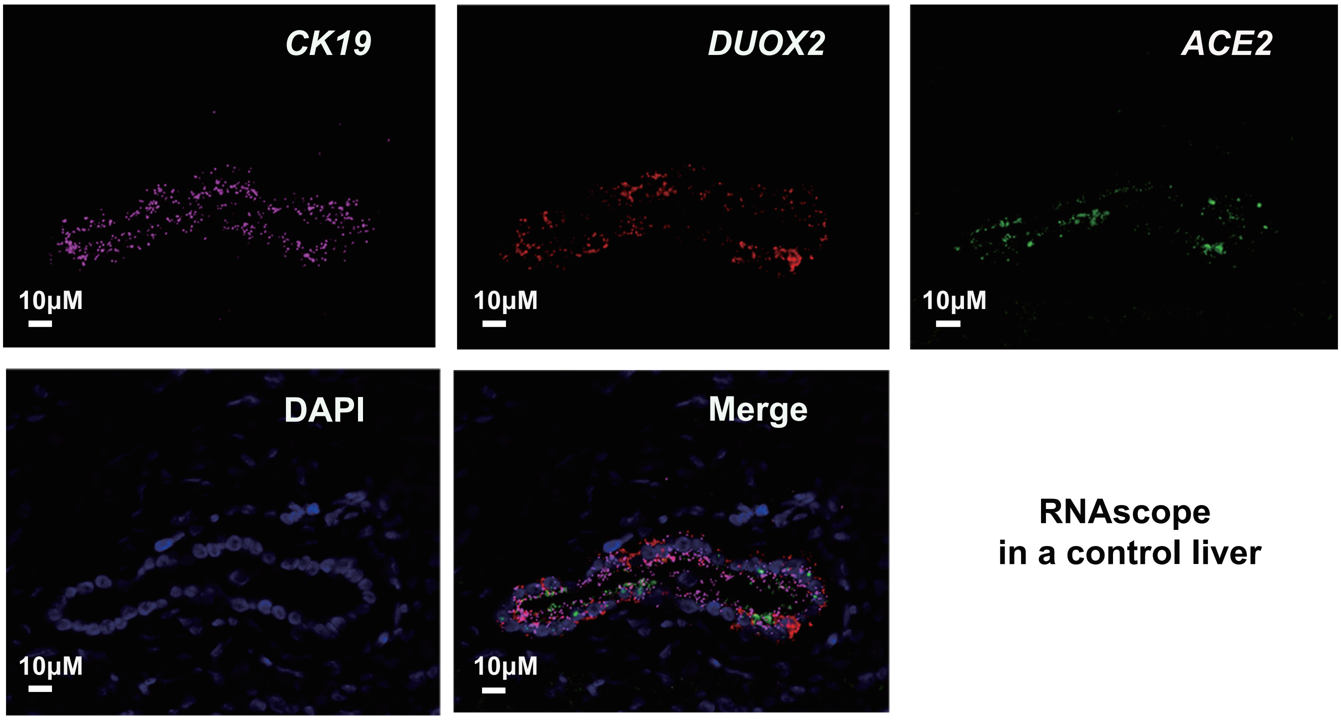

Supplementary Fig.13: Representative RNAscope photomicrographs of a bile duct containing both CK19$^+$DUOX2$^+$ACE2$^+$ cells and CK19$^+$DUOX2$^-$ACE2$^-$ cells in a control liver tissue section. The liver tissue section was stained using RNAscope with probes for *CK19* (purple), *DUOX2* (red) and *ACE2* (green). Scale bars: 10 μm. *n* = 3 independent experiments.

Supplementary Fig. 14

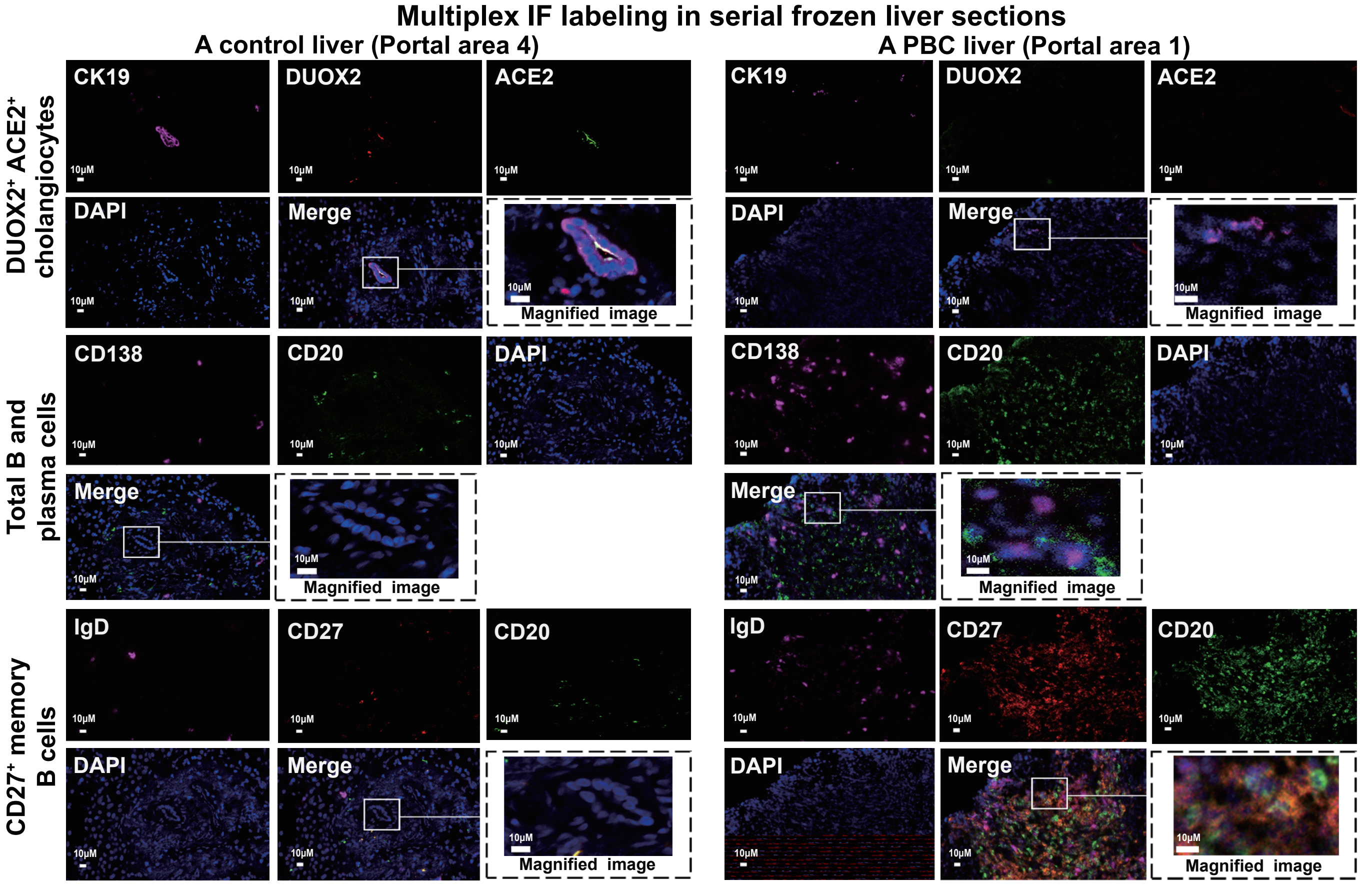

Supplementary Fig. 14: Representative multiplex immunofluorescence photomicrographs displayed the staining of each antibody in Fig.4f. Human serial frozen liver sections were analyzed by multiplex immunofluorescence (IF) with antibodies against CK19 (purple), DUOX2 (red), ACE2 (green), CD138 (purple), CD20 (green), IgD (purple) and CD27 (red). Scale bars:10 μm. $n = 3$ independent experiments.

Supplementary Fig. 15

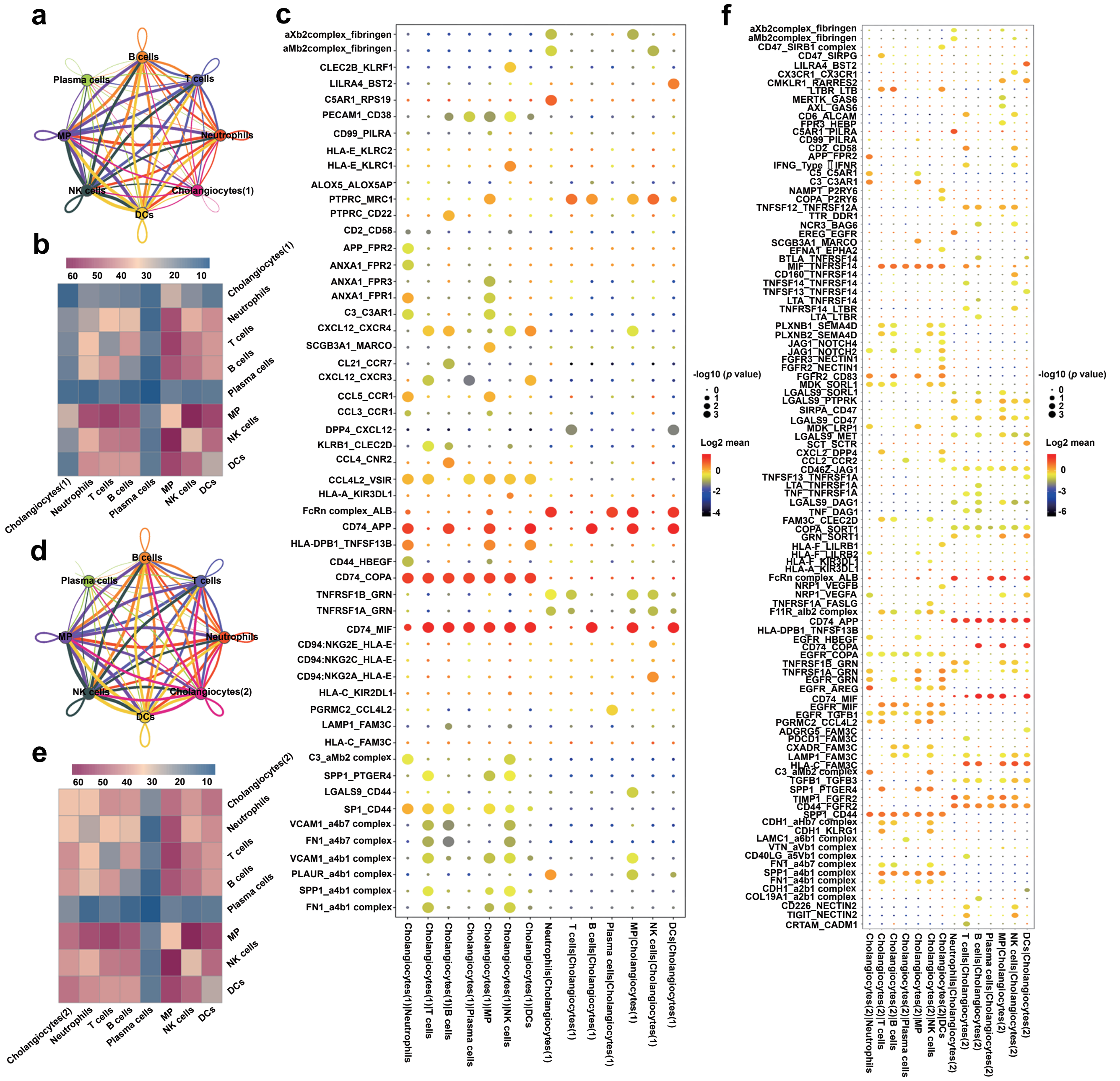

Supplementary Fig. 15: Interactions between different types of immune cells and the cluster (1) or (2) cholangiocytes. a Cellular interaction network displayed the potential interaction magnitude between the cluster (1) cholangiocytes and each type of immune cells in 4 control (CTR) and 5 primary biliary cholangitis (PBC) livers. Line thicknesses denote the numbers of ligand-receptor pairs. MP, mononuclear phagocytes; NK, natural killer cells; DCs, dendritic cells. **b** Heatmap displayed the total number of ligand-receptor pairs between the cluster (1) cholangiocytes and each type of immune cells. **c** Dot plot exhibited the significant ligand-receptor pairs involved in the interactions between the cluster (1) cholangiocytes and each type of immune cells. Ligand and cognate receptor are shown in the y axis; cell populations that express ligand and receptor are shown in the x axis. Circle sizes denote *p* values; colors (red, high; blue, low) denote average ligand and receptor expression levels in interacting subpopulations. Statistics: two-tailed permutation test without adjustment, 95% confidence interval. **d** Cellular interaction network predicted the potential interaction magnitude between the cluster (2) cholangiocytes and each type of immune cells in 4 CTR and 5 PBC livers as in **a**. **e** Heatmap displayed the total number of ligand-receptor pairs between the cluster (2) cholangiocytes and each type of immune cells. **f** Dot plot exhibited the significant ligand-receptor pairs involved in the interaction between the cluster (2) cholangiocytes and each type of immune cells as in **c**. Circle sizes denote *p* values; colors (red, high; blue, low) denote average ligand and receptor expression levels in interacting subpopulations. Statistics: two-tailed permutation test without adjustment, 95% confidence interval.

Supplementary Fig. 16

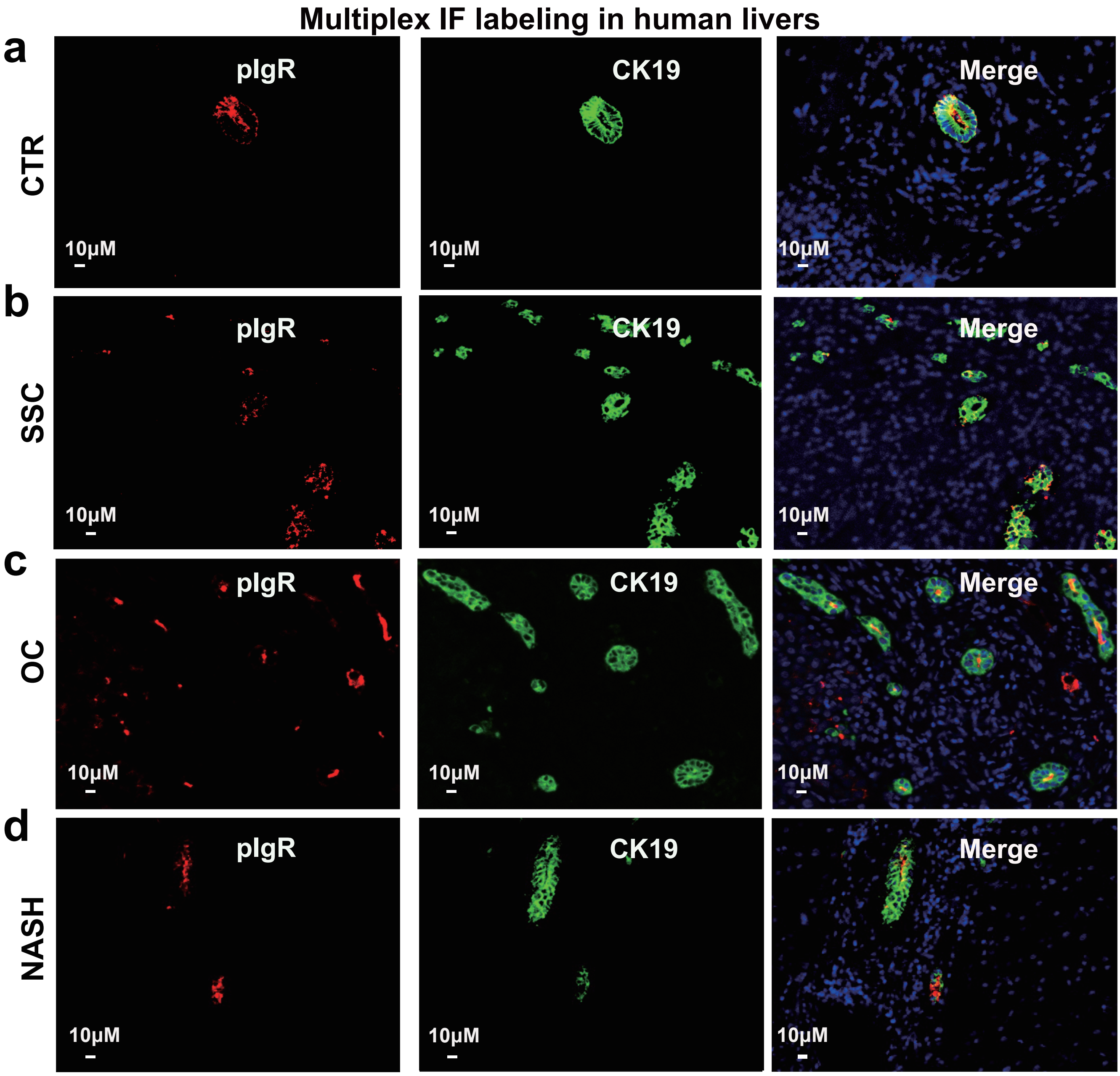

Supplementary Fig. 16: Representative multiplex immunofluorescence photomicrographs displayed pIgR and CK19 expression in human control and different disease status livers. a Representative multiplex immunofluorescence (IF) photomicrographs of a liver section from a control (CTR) patient after staining with antibodies against pIgR (red) and CK19 (green). Scale bars: 10 µm. *n* = 3 independent experiments. **b** Representative multiplex IF photomicrographs of a liver section from a secondary sclerosing cholangitis (SSC) patient after staining with antibodies against pIgR (red) and CK19 (green). Scale bars: 10µm. *n* = 3 independent experiments. **c** Representative multiplex IF photomicrographs of a liver section from a obstructive cholestasis (OC) patient after staining with antibodies against pIgR (red) and CK19 (green). Scale bars: 10 µm. *n* = 3 independent experiments**.** **d** Representative multiplex IF photomicrographs of a liver section from a nonalcoholic steatohepatitis (NASH) patient after staining with antibodies against pIgR (red) and CK19 (green). Scale bars: 10 µm. *n* = 3 independent experiments.

Supplementary Fig. 17

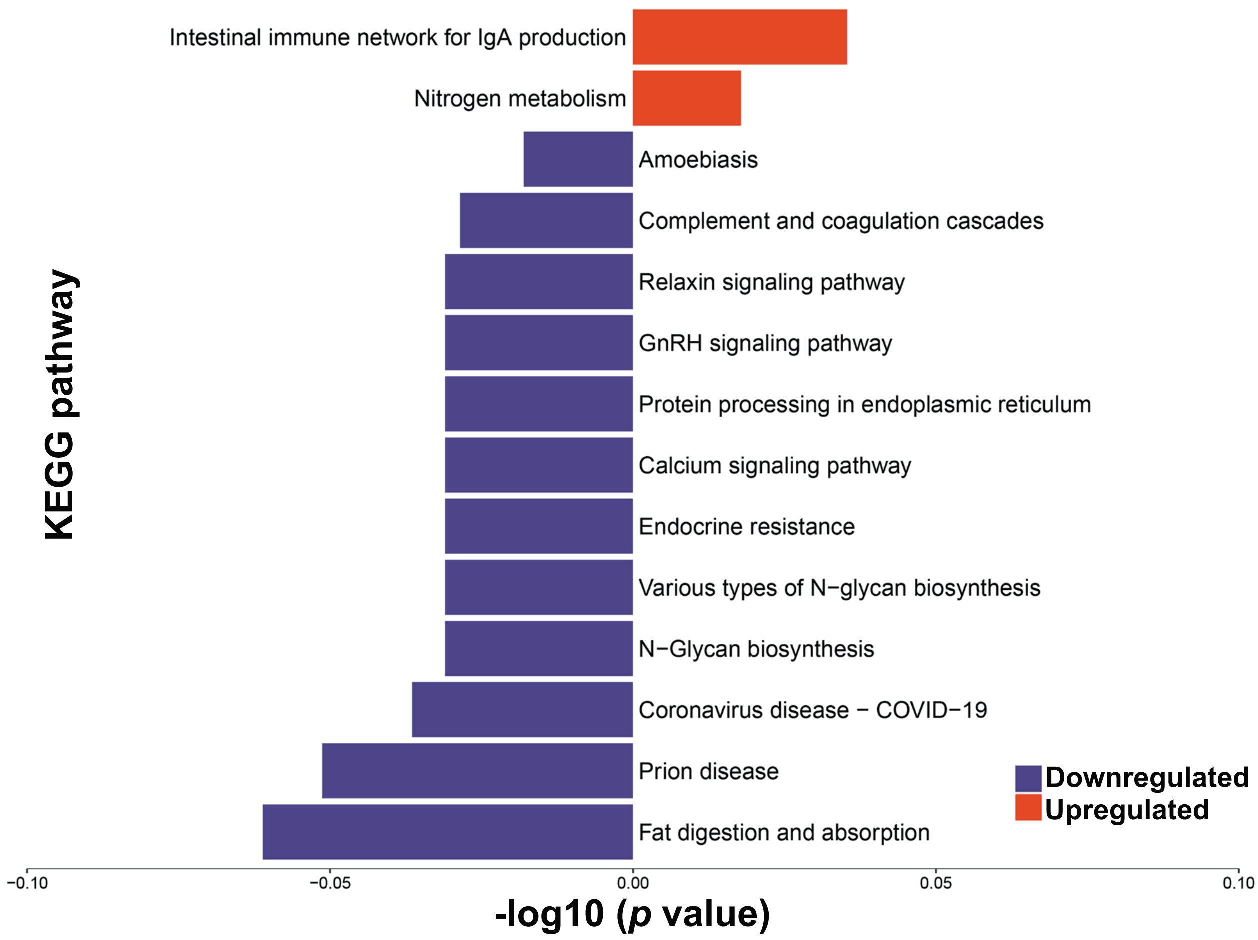

Supplementary Fig. 17: The intestinal immune network for the IgA production pathway was enriched by KEGG analysis of the proteomic data. Statistics: two-tailed Fisher's exact test without adjustment, 95% confidence interval.

Supplementary Fig. 18

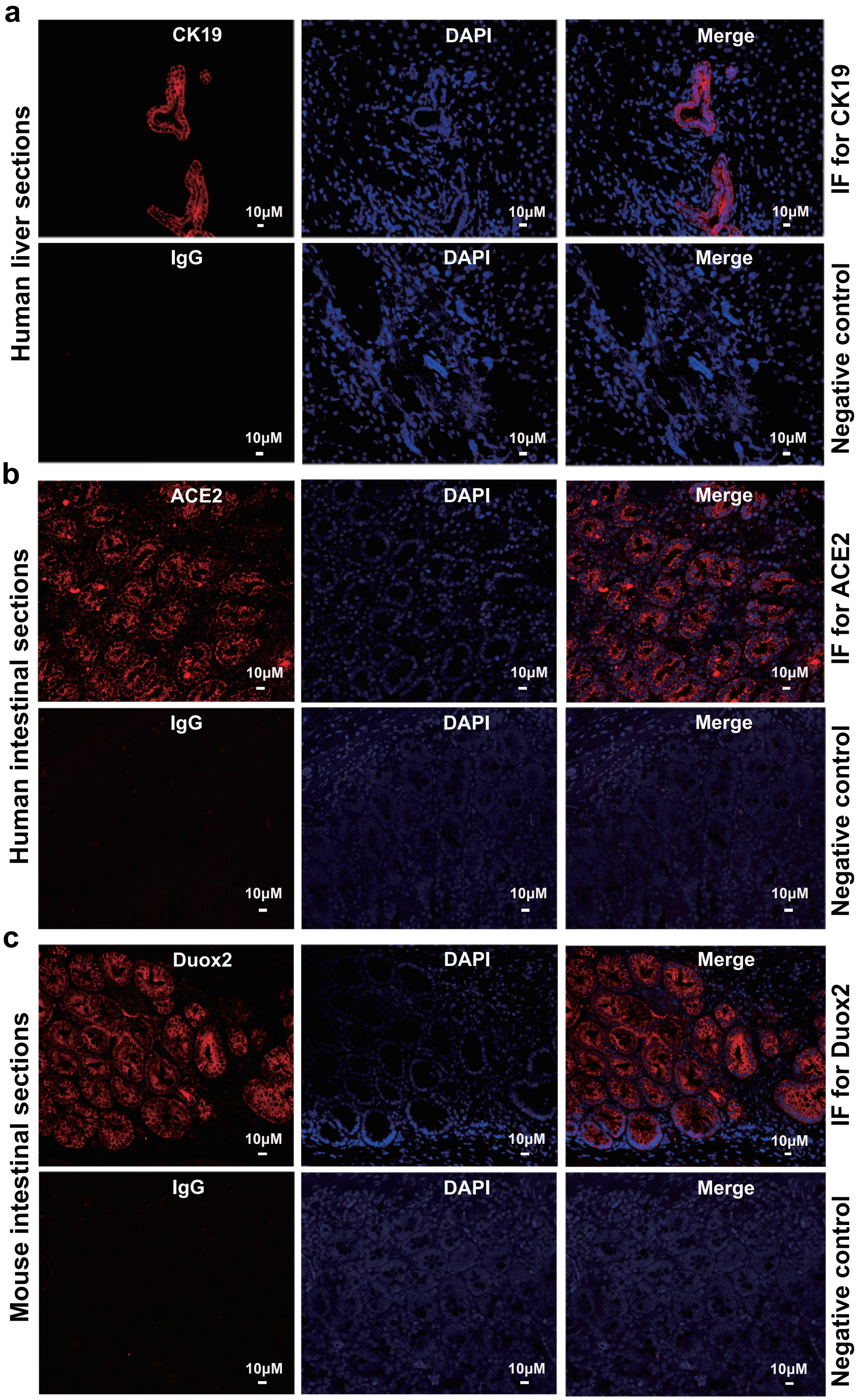

Supplementary Fig. 18. Validation the specificity of CK19, ACE2 and DUOX2 antibodies in the liver or intestinal sections. a Representative photomicrographs of human control liver tissue sections after multiplex immunofluorescence (IF) using antibodies against CK19 (red) or control IgG (negative control). Scale bars: 10 µm. *n* = 3 independent experiments. **b** Representative photomicrographs of human control intestines tissue sections after multiplex IF using antibodies against ACE2 (red) or control IgG (negative control). Scale bars: 10 µm. *n* = 3 independent experiments**. c** Representative photomicrographs of mouse normal intestines tissue sections after multiplex IF using antibodies against Duox2 (red) or control IgG (negative control). Scale bars: 10 µm. *n* = 3 independent experiments.

Supplementary Tables

Supplementary Table 1: The demographic and clinical characteristics of control and PBC patients for 5'- and 3'-scRNA-seq and ST analyses

**Characteristics**	**Ref.**	**Control patients**$^{\alpha}$	**PBC patients**$^{\alpha}$
Diagnosis, *n*	-	HH, 3; IHDS, 1	PBC, 5
Gender (F/M), *n*	-	3/1	4/1
Age (yrs)	-	52±7	55±4
BMI (kg/m^2)	-	23.5±1.7	21.8±1.2
ALT (IU/L)	0-42	21.3±5.8	92.9±25.2
AST (IU/L)	0-42	29.6±2.7	86.6±15.8
ALP (IU/L)	34-114	82.8±10.6*	262.4±58.3*
GGT (IU/L)	4-50	40.9±8.5*	447.9±204.6*
TBIL (μmol/L)	6-21	14.9±1.4	24.4±8.5
DBIL (μmol/L)	0-6	3.2±0.5	8.1±3.7
TBA (μmol/L)	0-10	5.5±1.0	33.3±23.9
ALB (g/L)	38-51	40.5±3.4	39.6±1.4
Cr (μmol/L)	45-84	56.9±3.0	58.1±1.2
PT (s)	9.8-13.7	11.6±0.4	10.5±0.4
PT-INR	0.9-1.2	1.0±0.04	0.9±0.04
WBC (×10^9/L)	3.5-9.5	5.0±1.0	4.9±0.6
HGB (g/L)	115-150	118.3±15.3	121.8±8.5
PLT (×10^9/L)	125-350	203.3±28.0	177.6±29.0

Abbreviations: PBC, primary biliary cholangitis; scRNA-seq, single-cell RNA sequencing; ST, spatial transcriptomics; Ref., reference; HH,

hepatic hemangioma; IHDS, intrahepatic duct stone; F, female; M, male; BMI, body mass index; ALT, alanine aminotransferase; AST, aspartate aminotransferase; ALP, alkaline phosphatase; GGT, gamma-glutamyl transferase; TBIL, total bilirubin; DBIL, direct bilirubin; TBA, total bile acids; ALB, albumin; Cr, creatinine; PT, prothrombin time; PT-INR, prothrombin time international normalized ratio; WBC, white blood cell count; HGB, hemoglobin; PLT, platelet count.

Notes: [α]Continuous data are expressed as mean ± standard error of mean; **p* = 0.016 obtained by two-tailed Mann-Whitney *U* test.

Supplementary Table 2: The detailed clinical characteristics of PBC patients for scRNA-seq and ST analyses

	PBC patients				
	PBC1	PBC2	PBC3	PBC4	PBC5
**Serum autoantibody**					
AMA-M2	+	+	+	+	+
ANA	N.D.	+	+	+	+
Anti-Sp100	-	-	-	-	N.D.
Anti-Gp210	-	-	-	-	N.D.
Anti-PML	-	-	-	-	N.D.
Anti-LKM-1	-	-	-	-	N.D.
Anti-LC-1	-	-	-	-	N.D.
Anti-SLA/LP	-	-	-	-	N.D.
Anti-Ro-52	+	+	+	-	-
Anti-U1RNP	-	N.D.	-	-	-
Anti-Sm	-	N.D.	-	-	-
Anti-SSA	+	N.D.	-	-	-
Anti-SSB	-	N.D.	-	-	-
Anti-Scl-70	-	N.D.	-	-	-
Anti-PM-Scl	-	N.D.	-	-	-
Anti-Jo-1	-	N.D.	-	-	+
Anti-CENP	-	N.D.	-	-	-
Anti-PCNA	-	N.D.	-	-	-
Anti-ds-DNA	-	N.D.	-	-	-
Anti-Nucleosome	-	N.D.	-	-	-
Anti-Histone	-	N.D.	-	-	-

Anti-Ribosome P protein	-	N.D.	-	-	-
**Serum immunoglobin, CER and AIH score**					
IgA (g/L)	6.1	1.2	1.4	2.3	1.3
IgG (g/L)	23.8	13.9	16.9	11.9	28.4
IgM (g/L)	9.5	4.5	6.2	1.9	2.9
IgE (g/L)	46.3	5.6	679.0	46.4	26.1
CER (mg/dl)	24.9	45.8	41.3	28.4	28.7
AIH score$^{\alpha}$	4	4	4	3	4
**Blood test for viral hepatitis**					
HAV IgM	-	-	-	-	-
HBsAg	-	-	-	-	-
HBV DNA	-	-	-	-	-
HCV Ag	-	-	-	-	-
HCV RNA	-	-	N.D.	-	-
HDV Ag	-	N.D.	-	-	N.D.
HDV IgG	-	N.D.	-	-	N.D.
HEV IgM	-	-	-	-	-
CMV DNA	-	-	N.D.	-	N.D.
**Fibrosis evaluating indicator**					
APRI	1.3	1.9	1.1	1.8	0.4
FIB-4	4.5	3.2	2.9	2.9	1.8
Child-Pugh class	A	A	A	A	A
Mayo risk score	3.9	3.6	4.4	5.6	4.6
**Histologic stage**					
Nakanuma stage	II	II	II	II	II

Fibrosis score	2	1	2	1	2
Bile duct loss score	0	0	0	0	0
CA grade	3	3	1	1	3
HA grade	2	2	3	2	3
Ludwing stage	III	II	III	II	II
**Clinical stage**	III	II	II	III	II

Abbreviations: PBC, primary biliary cholangitis; scRNA-seq, single-cell RNA sequencing; ST, spatial transcriptomics; AMA-M2, anti-mitochondrial antibody subtype M2; ANA, anti-nuclear antibody; Anti-PML, anti-promyelocytic leukemia protein; Anti-LKM-1, anti-liver kidney microsomal antibody type 1; Anti-LC-1, anti-lung cancer antigen-1; Anti-SLA/LP, anti-soluble liver antigen/liver pancreas; Anti-Ro-52, anti-52 kDa Ro protein; Anti-U1RNP, anti-U1 ribonucleoprotein; Anti-Sm, anti-smith; Anti-SSA, anti-Sjögren's syndrome A; Anti-SSB, anti-Sjögren's syndrome B; Anti-Scl-70, anti-scleroderma-70; Anti-PM-Scl, anti-polymyositis systemic-sclerosis overlap syndrome; Anti-CENP, anti-centromere protein; Anti-PCNA, anti-proliferating cell nuclear antigen; Anti-ds-DNA, anti-double stranded deoxyribonucleic acid; IgA, immunoglobulin A; IgG, immunoglobulin G; IgM, immunoglobulin M; IgE, immunoglobulin E; CER, ceruloplasmin; AIH, autoimmune hepatitis; HAV, hepatitis A virus; HBsAg, hepatitis B surface antigen; HBV, hepatitis B virus; HCV Ag, hepatitis C virus antigen; HDV, hepatitis D virus; HEV, hepatitis E virus; EBV, epstein-barr virus; CMV, cytomegalovirus; APRI, aspartate aminotransferase-to-platelet ratio index; FIB-4, fibrosis-4 index; CA, cholangitis activity; HA, hepatitis activity; N.D., not detect.

Notes: "-" denotes negative; "+" denotes positive; $^{\alpha}$According to the simplified criteria for the diagnosis of AIH (Scores ≥ 6, probable AIH; Scores ≥ 7, definite AIH)[1]

Supplementary Table 3: Quality metrics for scRNA-seq datasets

Sample	Pre-QC[α] (mean±S.E.M.)			QC[β]		Post-QC[γ] (mean±S.E.M.)			Cell number
	nGene	nUMI	Fraction.mito	nGene < 200	Fraction.mito > 0.5	nGene	nUMI	Fraction.mito	
CTR1	1335±14	3389±68	7.322±0.171	0.0117	0.0130	994±10	2517±33	6.384±0.096	3612
CTR2	1023±10	2764±46	14.251±0.149	0.0086	0.0282	866±7	1848±22	12.631±0.092	6883
CTR3	900±8	2174±33	11.993±0.088	0.0027	0.0076	772±5	1567±15	11.895±0.072	7960
CTR4	833±6	1869±24	13.582±0.063	0.0012	0.0013	707±4	1366±11	13.989±0.061	11323
PBC1	940±6	2331±36	9.481±0.089	0.0075	0.0073	831±4	1596±10	9.079±0.065	9373
PBC2	1562±8	4250±36	8.592±0.110	0.0107	0.0097	1471±6	3662±19	7.986±0.071	7948
PBC3	1151±7	3434±46	3.891±0.059	0.0040	0.0043	996±5	2227±19	3.594±0.033	10787
PBC4	1147±8	2469±29	8.274±0.129	0.0078	0.0125	1064±6	2072±14	7.405±0.076	5956
PBC5	628±6	1942±40	5.327±0.056	0.0198	0.0013	534±3	1207±13	5.454±0.046	6208

Abbreviations: QC, quality control; S.E.M., standard error of mean; nGene, number of genes; nUMI, number of unique molecular identifiers; fraction.mito, mitochondrial gene fraction; CTR, control patient; PBC, primary biliary cholangitis.

Notes: [α]Data prior to removal of poor quality cells; [β]The fraction of cells removed from each dataset as poor quality (nGene < 200 or fraction. mito > 0.5); [γ] Data after removal of poor quality cells.

Supplementary Table 4: The canonical marker genes for cell lineages

Cell lineage	Marker gene
Endothelial cells	*CDH5*$^+$*CLDN5*$^+$*KDR*$^+$
Cholangiocytes	*TM4SF4*$^+$*ANXA4*$^+$*KRT19*$^+$
Hepatocytes	*ALB*$^+$*HP*$^+$*TTR*$^+$*APOC1*$^+$
Mesenchymal cells	*RGS5*$^+$*ACTA2*$^+$*PDGFRB*$^+$*DCN*$^+$*LUM*$^+$*COL1A1*$^+$
Neutrophils	*LYZ*$^+$*S100A8*$^+$*S100A9*$^+$*CSF3R*$^+$*G0S2*$^+$
T cells	*CD3D*$^+$*CD3E*$^+$*CD3G*$^+$*TRBC2*$^+$*TRAC*$^+$
B cells	*MS4A1*$^+$*CD79A*$^+$*CD79B*$^+$
Plasma cells	*CD79A*$^+$*CD79B*$^+$*JCHAIN*$^+$*MZB1*$^+$
Mononuclear phagocytes	*LYZ*$^+$*CSF1R*$^+$*CD68*$^+$*MARCO*$^+$
Natural killer cells	*KLRD1*$^+$*NKG7*$^+$*XCL1*$^+$
Dendritic cells	*FCER1A*$^+$ *CLEC4C*$^+$*IL3RA*$^+$

Supplementary Table 5: The distribution of different types of cells in liver cells identified by 5'-scRNA-seq

**Cell subtype**	**CTR group**	**PBC group**
	**No. (%)**	**No. (%)**
Cholangiocytes	1653 (5.55%)	556 (1.38%)
Hepatocytes	8553 (28.72%)	9817 (24.38%)
Endothelial cells	2773 (9.31%)	964 (2.39%)
Mesenchymal cells	3346 (11.24%)	491 (1.22%)
T cells	6644 (22.31%)	18047 (44.81%)
B cells	254 (0.85%)	2379 (5.91%)
Plasma cells	115 (0.39%)	929 (2.31%)
Natural killer cells	1758 (5.90%)	3740 (9.29%)
Mononuclear phagocytes	4400 (14.78%)	3285 (8.16%)
Dendritic cells	41 (0.14%)	60 (0.15%)
Neutrophils	241 (0.81%)	4 (0.01%)
Total	29778	40272

Abbreviations: CTR, control patient; PBC, primary biliary cholangitis; No., number. Source data are provided as a Source Data file.

Supplementary Table 6: The distribution of cholangiocyte subpopulations in each group identified by 5’-scRNA-seq

Subpopulations of cholangiocytes	CTR group	PBC group
	No. (%)	No. (%)
Cholangiocytes(1)	707 (42.77%)	201 (36.15%)
Cholangiocytes(2)	541 (32.73%)	147 (26.44%)
Cholangiocytes(3)	308 (18.63%)	0 (0)
Cholangiocytes(4)	13 (0.79%)	76 (13.67%)
Cholangiocytes(5)	46 (2.78%)	32 (5.76%)
Cholangiocytes(6)	0 (0)	52 (9.35%)
Cholangiocytes(7)	9 (0.54%)	41 (7.37%)
Cholangiocytes(8)	29 (1.75%)	7 (1.26%)
Total	1653	556

Abbreviations: CTR, control patient; PBC, primary biliary cholangitis; No., number. Source data are provided as a Source Data file.

Supplementary Table 7: The proportions of the cluster 3 cholangiocytes in each subject from 5'-scRNA-seq data

**Proportion[α]**	**CTR1**	**CTR2**	**CTR3**	**CTR4**	**PBC1**	**PBC2**	**PBC3**	**PBC4**	**PBC5**
Cholangiocytes(3) (%)	25.74	7.55	3.93	22.25	0	0	0	0	0

Abbreviation：CTR, control patient; PBC, primary biliary cholangitis.

Notes: [α]The proportion of DUOX2$^+$ACE2$^+$ cholangiocytes in all cholangiocytes for each patient. Source data are provided as a Source Data file.

Supplementary Table 8: Clinical characteristics of the studied subjects for FACS analysis

ID	Sample type	Application[α]	Gender, (F/M), *n*	Age (yrs)[β]	ALT (IU/L)	AST (IU/L)	ALP (IU/L)	GGT (IU/L)	TBIL (μmol/L)	Diagnosis
C1	Liver	Cholangiocytes			10.7	13.4	58.0	9.8	8.7	HH
C2	Liver	Cholangiocytes	1/2	42±6	51.1	24.1	51.0	33.1	12.5	HH
C3	Liver	Cholangiocytes			57.6	50.8	60.0	17.1	15.7	HH

Abbreviations: FACS, fluorescence-activated cell sorting; F, female; M, male; ALT, alanine aminotransferase; AST, aspartate aminotransferase; ALP, alkaline phosphatase; GGT, gamma-glutamyl transferase; TBIL, total bilirubin; HH, hepatic hemangioma.

Notes: [α]Sorted cell type for fluorescence-activated cell sorting. [β]Expressed as mean ± standard error of mean.

Supplementary Table 9: Clinical characteristics of the studied subjects for multiplex immunofluorescence and RNAscope

Group	Gender (F/M), *n*	Age (yrs) $^{\alpha}$	ID$^{\beta}$	AMA-M2	Ludwing stage	Nakanuma stage	Fibrosis score	BD loss score	CA grade	HA grade	Cell percent (%)$^{\gamma}$
PBC	15/3	54±2	P(1)	+	III	III	3	0	3	3	13.9
			P(2)	+	II	II	2	0	3	2	16.3
			P(3)	-	III	III	3	0	2	0	9.5
			P(4)	-	II	II	2	0	1	2	13.3
			P(5)	+	III	III	3	0	3	3	2.7
			P(6)	N.D.	II	II	2	0	1	1	11.0
			P(7)	+	III	III	3	1	3	3	3.9
			P(8)	+	III	III	3	0	3	3	10.5
			P(9)	+	III	III	3	1	2	3	5.5
			P(10)	+	II	II	2	0	1	1	11.4
			P(11)	+	II	III	2	1	2	0	11.0
			P(12)	+	II	II	2	0	2	1	15.1
			P(13)	-	II	II	2	0	2	2	7.1
			P(14)	-	III	III	3	0	3	3	0.8
			P(15)	-	III	II	2	0	3	3	8.4
			P(16)	+	II	II	2	0	3	1	N.D.
			P(17)	+	II	II	2	0	3	3	N.D.
			P(18)	+	N.D.	N.D.	N.D.	N.D.	N.D.	N.D.	N.D.
			C(1)	N.D.	N.D.	N.D.	N.D.	N.D.	N.D.	N.D.	33.8
			C(2)	N.D.	N.D.	N.D.	N.D.	N.D.	N.D.	N.D.	19.7
			C(3)	N.D.	N.D.	N.D.	N.D.	N.D.	N.D.	N.D.	21.6
			C(4)	N.D.	N.D.	N.D.	N.D.	N.D.	N.D.	N.D.	15.0

			C(5)	N.D.	N.D.	N.D.	N.D.	N.D.	N.D.	N.D.	39.7
			C(6)	N.D.	N.D.	N.D.	N.D.	N.D.	N.D.	N.D.	28.3
			C(7)	N.D.	N.D.	N.D.	N.D.	N.D.	N.D.	N.D.	33.3
Control	12/5	41±3	C(8)	N.D.	N.D.	N.D.	N.D.	N.D.	N.D.	N.D.	32.7
			C(9)	N.D.	N.D.	N.D.	N.D.	N.D.	N.D.	N.D.	27.8
			C(10)	N.D.	N.D.	N.D.	N.D.	N.D.	N.D.	N.D.	N.D.
			C(11)	N.D.	N.D.	N.D.	N.D.	N.D.	N.D.	N.D.	N.D.
			C(12)	N.D.	N.D.	N.D.	N.D.	N.D.	N.D.	N.D.	N.D.
			C(13)	N.D.	N.D.	N.D.	N.D.	N.D.	N.D.	N.D.	N.D.
			C(14)	N.D.	N.D.	N.D.	N.D.	N.D.	N.D.	N.D.	N.D.
			C(15)	N.D.	N.D.	N.D.	N.D.	N.D.	N.D.	N.D.	N.D.
			C(16)	N.D.	N.D.	N.D.	N.D.	N.D.	N.D.	N.D.	N.D.
			C(17)	N.D.	N.D.	N.D.	N.D.	N.D.	N.D.	N.D.	N.D.
			N(1)	N.D.	N.D.	N.D.	N.D.	N.D.	N.D.	N.D.	N.D.
Others	3/0	48±6	O(1)	N.D.	N.D.	N.D.	N.D.	N.D.	N.D.	N.D.	N.D.
			S(1)	N.D.	N.D.	N.D.	N.D.	N.D.	N.D.	N.D.	N.D.

Abbreviations: F, female; M, male; AMA-M2, anti-mitochondrial antibody subtype M2; BD, bile duct; CA, cholangitis activity; HA, hepatitis activity; PBC, primary biliary cholangitis; N.D., not detect.

Notes:$^{\alpha}$Expressed as mean ± standard error of mean. $^{\beta}$P(1)-(17), PBC patients whose liver tissue samples used for multiplex immunofluorescence; P(18), a PBC patient whose liver tissue sample used for RNAscope; C(1)-(12), control patients whose liver tissue samples used for multiplex immunofluorescence, including C(1) with hepatic papillary adenoma, C(2) with intrahepatic bile duct stones, C(3) with biliary anastomotic stricture, C(4) with inflammatory myofibroblastoma of hepatogastric space, C(5) with cholangiocarcinoma,

C(6) with intrahepatic bile duct stones, C(7) with periampullary carcinoma, C(8) with pancreatic adenocarcinoma, C(9) with pancreatic papilloma, and C(10)-C(12) with hepatic hemangioma; C(13), a patient with colonic polyp whose intestinal tissue sample used for multiplex immunofluorescence; C(12) and C(14)-(17), control patients with hepatic hemangioma whose liver tissue samples used for RNAscope; N(1), O(1) and S(1), patients with nonalcoholic steatohepatitis, obstructive cholestasis and secondary sclerosing cholangitis respectively, whose liver tissue samples used for multiplex immunofluorescence. [γ]The precent of DUOX2$^+$ACE2$^+$ small cholangioctytes in all cholangiocytes. “-” denotes negative; “+” denotes positive.

Supplementary Table 10: Clinical characteristics of the studied subjects for serum proteomic analysis

Group	Gender (F/M), *n*	Age (yrs)[α]	ID	Sample type	AMA-M2
Healthy	5/1	27±1	H1	Serum	N.D.
			H2	Serum	N.D.
			H3	Serum	N.D.
			H4	Serum	N.D.
			H5	Serum	N.D.
			H6	Serum	N.D.
PBC	8/2	58±3	P(a1)	Serum	+
			P(a2)	Serum	+
			P(a3)	Serum	+
			P(a4)	Serum	-
			P(a5)	Serum	-
			P(a6)	Serum	+
			P(a7)	Serum	+
			P(a8)	Serum	+
			P(a9)	Serum	+
			P(a10)	Serum	+

Abbreviations: F, female; M, male; AMA-M2, anti-mitochondrial antibody subtype M2; PBC, primary biliary cholangitis; N.D., not detect.

Notes: [α]Expressed as mean ± standard error of mean. "-" denotes negative; "+" denotes positive.

Supplementary Table 11: Serum pIgR protein abundance in PBC patients and healthy controls obtained by proteomics

Patient	P(a1)	P(a2)	P(a3)	P(a4)	P(a5)	P(a6)	P(a7)	P(a8)	P(a9)	P(a10)
pIgR	2585.87	516.45	632.33	374.35	1926.17	618.90	744.43	387.87	2666.69	527.51
**Control**	**H1**	**H2**	**H3**	**H4**	**H5**	**H6**				
pIgR	115.90	197.80	237.68	268.11	410.15	180.53				

Notes: P(a1)-(a10), PBC patients; H1-6, healthy volunteers.

Supplementary Table 12: Clinical characteristics of the studied subjects for ELISA detection of serum anti-pIgR antibodies

Group	Gender (F/M), *n*	Age (yrs)[α]	ID[β]	Sample type	AMA-M2
			P(b1)	Serum	+
			P(b2)	Serum	+
			P(b3)	Serum	+
			P(b4)	Serum	+
			P(b5)	Serum	-
			P(b6)	Serum	-
			P(b7)	Serum	+
			P(b8)	Serum	+
PBC	14/3	55±2	P(b9)	Serum	+
			P(b10)	Serum	+
			P(b11)	Serum	+
			P(b12)	Serum	+
			P(b13)	Serum	+
			P(b14)	Serum	+
			P(b15)	Serum	+
			P(b16)	Serum	+
			P(b17)	Serum	+
			C(b1)	Serum	N.D.
			C(b2)	Serum	N.D.
			C(b3)	Serum	N.D.
			C(b4)	Serum	N.D.
			C(b5)	Serum	N.D.
Control	9/3	46±3	C(b6)	Serum	N.D.

			C(b7)	Serum	N.D.
			C(b8)	Serum	N.D.
			C(b9)	Serum	N.D.
			C(b10)	Serum	N.D.
			C(b11)	Serum	N.D.
			C(b12)	Serum	N.D.
			O1	Serum	N.D.
			O2	Serum	N.D.
			O3	Serum	N.D.
			O4	Serum	N.D.
			O5	Serum	N.D.
			O6	Serum	N.D.
			O7	Serum	N.D.
OC	5/10	55±3	O8	Serum	N.D.
			O9	Serum	N.D.
			O10	Serum	N.D.
			O11	Serum	N.D.
			O12	Serum	N.D.
			O13	Serum	N.D.
			O14	Serum	N.D.
			O15	Serum	N.D.

Abbreviations: ELISA, enzyme linked immunosorbnent assay; pIgR, polymeric immunoglobulin receptor; PBC, primary biliary cholangitis; F, female; M, male; AMA-M2, anti-mitochondrial antibody subtype M2; N.D., not detect; OC, obstructive cholestasis.

Notes: $^{\alpha}$Expressed as mean ± standard error of mean. $^{\beta}$C(b1)-C(b12) patients without cholestasis.

Supplementary Table 13: The canonical marker genes for cell subtypes

Cell lineages	Cell subtypes	Marker genes
Endothelial cells		
	LEC	*PDPN*$^+$*PROX1*$^+$*LYVE1*$^+$
	PVEC	*CPE*$^+$*CD320*$^+$
	HAEC	*CD34*$^+$*AIF1L*$^+$*SOX17*$^+$
	SEC	*CLEC4G*$^+$*CLEC4M*$^+$*LYVE1*$^+$
	CVEC	*RSPO3*$^+$*WNT2*$^+$*SELE*$^+$*SELP*$^+$
Mesenchymal cells		
	Fibroblast	*DCN*$^+$*LUM*$^+$*COL3A1*$^+$*COL1A1*$^+$
	HSC	*RGS5*$^+$*PDGFRB*$^+$
	VSMC	*MYH11*$^+$*ACTA2*$^+$
T + NK cells		
	Naive CD4	*CD4*$^+$*CCR7*$^+$*IL7R*$^+$*LEF1*$^+$*TCF7*$^+$*SELL*$^+$
	Th1	*CD4*$^+$*CXCR3*$^+$*IFNG*$^+$*GZMA*$^+$*GZMK*$^+$
	Th17	*CD4*$^+$*CCR6*$^+$*RORA*$^+$*RORC*$^+$*IL23R*$^+$*CCR4*$^+$
	Tfh	*TCF7*$^+$*BCL6*$^+$*CXCR5*$^+$*CXCL13*$^+$*PDCD1*$^+$*CD200*$^+$*ICOS*$^+$*CD40LG*$^+$
	Treg	*CD3D*$^+$*FOXP3*$^+$*IL2RA*$^+$*CTLA4*$^+$*IKZF2*$^+$*TNFRSF4*$^+$
	Naive CD8	*CD8A*$^+$*CD8B*$^+$*CCR7*$^+$*IL7R*$^+$*LEF1*$^+$*TCF7*$^+$*SELL*$^+$
	CD8&γδT_Tscm	*CD8A*$^+$*CXCR3*$^+$*TRDC*$^+$*XCL1*$^+$*XCL2*$^+$*TBX21*$^+$
	CX3CR1$^+$CD8 Teff	*CD8A*$^+$*CX3CR1*$^+$*TBX21*$^+$*GZMB*$^+$*PRF1*$^+$
	CD8 Trm	*CD8A*$^+$*ITGA1*$^+$*STAT4*$^+$*IFNG*$^+$*CD69*$^+$
	CD8 exhausted T	*CD3D*$^+$*CD8A*$^+$*LAG3*$^+$*PDCD1*$^+$*CTLA4*$^+$
	Mitotic CD8	*CD8*$^+$*AMKI67*$^+$*ITGAE*$^+$
	MAIT	*TRAV1-2*$^+$*KLRB1*$^+$*MAF*$^+$*IL23R*$^+$

	Cir-NK	$KLRD1^{+}NCAM1^{dim}CX3CR1^{+}$
	lr-NK	$KLRD1^{+}NCAM1^{+}CX3CR1^{+}CD69^{+}$
	LTi	$SELL^{+}KLRD1loIL7R^{+}TCF7^{+}KIT^{+}AHR^{+}LTB^{+}$
B + Plasma cells		
	Naive B	$CD19^{+}CD27^{-}IGHD^{+}$
	$CD27^{-}$ memory B	$CD27^{-}$
	$CD27^{+}$memory B	$CD27^{+}$
	PB	$CD27^{+}CD38^{+}MKI67^{+}$
	PC	$CD27^{+}$ $CD38^{+}TNFRSF17^{+}$
MP + DC		
	$CD14^{+}$ Mo	$LYZ^{+}CD68^{+}CD14^{+}S100A8^{+}S100A9^{+}$
	$CD14^{+}CD16^{+}$Mo	$LYZ^{+}CD68^{+}CD14^{+}FCGR3A^{+}S100A8^{+}S100A9^{+}$
	$CD16^{+}$ Mo	$LYZ^{+}CD68^{+}FCGR3A^{+}S100A8^{+}S100A9^{+}$
	Kupffer cells	$CD14^{+}FCGR3A^{+}C1QB^{+}MARCO^{+}CD5L^{+}CD163^{+}$
	cDC1	$XCR1^{+}CLEC9A^{+}THBD^{+}$
	cDC2	$CD1C^{+}FCER1A^{+}$
	pDC	$LILRA4^{+}IRF4^{+}CLEC4C^{+}IL3RA^{+}$
	Mature DC	$CCR7^{+}CCL19^{+}LAMP3^{+}NCCRP1^{+}LAD1^{+}$
	Mitotic myeloid	$MKI67^{+}PCNA^{+}$

Abbreviations: LEC, lymphatic endothelial cells; PVEC, portal vein endothelial cells; HAEC, hepatic artery endothelial cells; SEC, sinusoidal endothelial cells; CVEC, central vein endothelial cells; HSC, hepatic stellate cells; VSMC, vascular smooth muscle cells; Tscm, T memory stem cells; Teff, effector T cells; Trm, tissue resident memory T cells; MAIT, mucosal-associated invariant T cells; Th1, T helper 1 cells; Th17, T helper 17 cells; Tfh, follicular helper T cells; Treg, regulatory T cells; Cir-NK, circulating NK cells; lr-NK, liver-resident NK cells; LTi, lymphoid tissue inducer cells; PB, plasmablasts; PC, plasma cells; Mo, monocytes; cDC1, conventional type 1 dendritic cells; cDC2, conventional type 2 dendritic cells; pDC, plasmacytoid dendritic cells; Mature DC, mature dendritic cells.

Supplementary Table 14: Antibodies used in the study

Name	Conjugate	Target	Host	Company / Catalog	Application	Dilution
Ace2	-	Mouse	Rabbit	Abcam, Cambridge, MA/ab108252	IF	1:500
Duox2	-	Mouse	Rabbit	Abcam, Cambridge, MA/ab97266	IF	1:500
Ck19	-	Mouse	Rabbit	Abcam, Cambridge, MA/ab52625	IF	1:500
pIgR	-	Mouse	Rabbit	Proteintech, Chicago, IL/22024-1-AP	IF	1:500
Ck7	-	Mouse	Rabbit	Abcam, Cambridge, MA/ab181598	IF	1:100
Rabbit IgG	-	Mouse	Rabbit	Proteintech, Chicago, IL/B900610	IF	1:500
Mouse IgG	-	Mouse	Mouse	Proteintech, Chicago, IL/B900620	IF	1:500
CD20	-	Human	Rabbit	ZSGB-BIO, Beijing, BJ/ZA-0549	IF	1:250
CD27	-	Human	Mouse	Proteintech, Chicago, IL/66308-1-Ig	IF	1:500
IgD	-	Human	Rabbit	ZSGB-BIO, Beijing, BJ/ZA-0443	IF	1:75
CD138	-	Human	Rabbit	ZSGB-BIO, Beijing, BJ/ZA-0584	IF	1:200
ACE2	-	Human	Mouse	Proteintech, Chicago, IL/66699-1-Ig	IF	1:2000
DUOX2	-	Human	Rabbit	Abcam, Cambridge, MA/ab97266	IF	1:500
CK19	-	Human	Rabbit	Abcam, Cambridge, MA/ab52625	IF	1:500
pIgR	-	Human	Rabbit	Proteintech, Chicago, IL/22024-1-AP	IF	1:500
CK7	-	Human	Rabbit	Abcam, Cambridge, MA/ab181598	IF	1:100
Rabbit IgG	-	Human	Rabbit	Proteintech, Chicago, IL/B900610	IF	1:500
Mouse IgG	-	Human	Mouse	Proteintech, Chicago, IL/B900620	IF	1:2000
Duox2	-	Mouse	Mouse	Santa cruz, Dallas, TX/sc-398681	FACS	1:50
Ck19	Alexa Fluor® 647	Mouse	Rabbit	Abcam, Cambridge, MA/ab192980	FACS	1:50
Ace2	CoraLite®488 Fluorescent Dye	Mouse	Mouse	Proteintech,Chicago, IL/CL488-66699	FACS	1:50
IgG2a heavy chain	PE/Cy7®	Mouse	Goat	Abcam, Cambridge, MA/ab130787	FACS	1:50

DUOX2	-	Human	Mouse	Santa cruz, Dallas, TX/sc-398681	FACS	1:50
CK19	Alexa Fluor® 647	Human	Rabbit	Abcam, Cambridge, MA/ab192980	FACS	1:50
ACE2	CoraLite®488 Fluorescent Dye	Human	Mouse	Proteintech, Chicago, IL/CL488-66699	FACS	1:50
IgG2a heavy chain	PE/Cy7®	Human	Goat	Abcam, Cambridge, MA/ab130787	FACS	1:50
Isotype control to DUOX2	PE/Cy™7	Human/Mouse	Mouse	BD Biosciences, Franklin Lakes, NJ/557872	FACS	1:50
Isotype control to CK19	Alexa Fluor® 647	Human/Mouse	Rabbit	Abcam, Cambridge, MA/ab199093	FACS	1:50
Isotype control to ACE2	CoraLite®488	Human/Mouse	Mouse	Proteintech, Chicago, IL/ CL488-65124	FACS	1:50
CK7	-	Human	Rabbit	Abcam, Cambridge, MA/ab181598	IHC	1:8000

Abbreviations: IF, immunofluorescence; FACS, fluorescence-activated cell sorting; IHC, immunohistochemistry.

Supplementary Table 15: The sequences of RT-qPCR primers

Gene	Sequences (5'→3')	Species/Source
Gapdh	Forward: 5'- ccaaggtcatccatgacaac-3'	Mouse/Primers (SYBR)
	Reverse: 5'- tgtcataccaggaaatgagc-3'	NM_008084
Cftr	Forward: 5'- cccttcggcgatgctttttc-3'	Mouse/Primers (SYBR)
	Reverse: 5'- aagcctatgccaaggtaaatgg-3'	NM_021050
Sctr	Forward: 5'- gcccagattgtgtgatgtgc-3'	Mouse/Primers (SYBR)
	Reverse: 5'- cggtgagaatacgatggctgat-3'	NM_001012322
Ck7	Forward: 5'- aggagatcaaccgacgcac-3'	Mouse/Primers (SYBR)
	Reverse: 5'- caccttgttcgtgtaggcg-3'	NM_033073
Sox9	Forward: 5'- agtacccgcatctgcacaac-3'	Mouse/Primers (SYBR)
	Reverse: 5'- acgaagggtctcttctcgct-3'	NM_011448
pIgR	Forward: 5'- atgaggctctacttgttcacgc-3'	Mouse/Primers (SYBR)
	Reverse: 5'- acctcctggggaccaaatatg-3'	NM_002644

Supplementary References

1. Galaski, J. et al. Update of the simplified criteria for autoimmune hepatitis: Evaluation of the methodology for immunoserological testing. *J. Hepatol*. **74**, 312-320 (2012)
