# Supplementary material for: Unique DUOX2+ACE2+ small cholangiocytes are pathogenic targets for primary biliary cholangitis
Source: Nat Commun. 2023 Feb 9;14:29. doi: 10.1038/s41467-022-34606-w (PMC9911648; doi:10.1038/s41467-022-34606-w)
Supplement: Supplementary file 2 — Description of Additional Supplementary Files [file 41467_2022_34606_MOESM2_ESM.pdf]

Description of Additional Supplementary Files

File name: Supplementary Data 1

Description: Differentially expressed genes of the clusters identified from 70,050 liver cells

File name: Supplementary Data 2

Description: Differentially expressed genes of the cell lineages identified from 70,050 liver cells

File name: Supplementary Data 3

Description: Differentially expressed genes of the clusters identified from 2,209 cholangiocytes

File name: Supplementary Data 4

Description: Differentially expressed genes of the clusters identified from 25,549 liver T cells and natural killer cells

File name: Supplementary Data 5

Description: Differentially expressed genes of the clusters identified from 2,783 liver B cells and plasma cells

File name: Supplementary Data 6

Description: Differentially expressed genes of the clusters identified from 4,873 liver mononuclear phagocytes and dendritic cells

File name: Supplementary Data 7

Description: Differentially expressed genes of the clusters identified from 2,664 liver endothelial cells

File name: Supplementary Data 8

Description: Differentially expressed genes of the clusters identified from 1,136 liver mesenchymal cells
