# Supplementary material for: Unique DUOX2+ACE2+ small cholangiocytes are pathogenic targets for primary biliary cholangitis
Source: Nat Commun. 2023 Feb 9;14:29. doi: 10.1038/s41467-022-34606-w (PMC9911648; doi:10.1038/s41467-022-34606-w)
Supplement: Supplementary file 11 — Reporting Summary [file 41467_2022_34606_MOESM11_ESM.pdf]

nature portfolio

Corresponding author(s): Jin Chai, Shifang Peng, Xiaowei Liu, Can-E Tang, Xuequan Huang

Last updated by author(s): Oct 31, 2022

Reporting Summary

Nature Portfolio wishes to improve the reproducibility of the work that we publish. This form provides structure for consistency and transparency in reporting. For further information on Nature Portfolio policies, see our Editorial Policies and the Editorial Policy Checklist.

Statistics

For all statistical analyses, confirm that the following items are present in the figure legend, table legend, main text, or Methods section.

n/a	Confirmed	
☐	☒	The exact sample size (*n*) for each experimental group/condition, given as a discrete number and unit of measurement
☐	☒	A statement on whether measurements were taken from distinct samples or whether the same sample was measured repeatedly
☐	☒	The statistical test(s) used AND whether they are one- or two-sided<br>*Only common tests should be described solely by name; describe more complex techniques in the Methods section.*
☒	☐	A description of all covariates tested
☒	☐	A description of any assumptions or corrections, such as tests of normality and adjustment for multiple comparisons
☐	☒	A full description of the statistical parameters including central tendency (e.g. means) or other basic estimates (e.g. regression coefficient) AND variation (e.g. standard deviation) or associated estimates of uncertainty (e.g. confidence intervals)
☐	☒	For null hypothesis testing, the test statistic (e.g. *F*, *t*, *r*) with confidence intervals, effect sizes, degrees of freedom and *P* value noted<br>*Give P values as exact values whenever suitable.*
☒	☐	For Bayesian analysis, information on the choice of priors and Markov chain Monte Carlo settings
☒	☐	For hierarchical and complex designs, identification of the appropriate level for tests and full reporting of outcomes
☒	☐	Estimates of effect sizes (e.g. Cohen's *d*, Pearson's *r*), indicating how they were calculated

Our web collection on statistics for biologists contains articles on many of the points above.

Software and code

Policy information about availability of computer code

Data collection	No software was used to collect data.
Data analysis	Software used for the analyses are: FastQC (version 0.11.4); Fastp (version 1); Cutadapt(version 1.17); STAR (version 2.5.3a); FeatureCounts (version 1.6.2); Seurat (version 3.1.2); Harmony (version 1.0); ClusterProfiler (version 4.4.4); R (version 3.6.3); Cellranger(version 3.1.0); CellPhoneDB (version 2.1.0); Circlize (version 0.4.15); StLearn (version 0.3.1); FlowJo (version 10.6.2); ZEN (version 2012); Spectronaut (version 14.4.200727.47784); Cluster 3.0 (version 3.0); Java Treeview (version 3.0); InterProScan (version 5.25-64.0); SPSS (version 23); GraphPad Prism (version 8).

For manuscripts utilizing custom algorithms or software that are central to the research but not yet described in published literature, software must be made available to editors and reviewers. We strongly encourage code deposition in a community repository (e.g. GitHub). See the Nature Portfolio guidelines for submitting code & software for further information.

Data

Policy information about availability of data

All manuscripts must include a data availability statement. This statement should provide the following information, where applicable:

- Accession codes, unique identifiers, or web links for publicly available datasets
- A description of any restrictions on data availability
- For clinical datasets or third party data, please ensure that the statement adheres to our policy

The raw data of scRNA-seq, BCR and TCR sequencing, and spatial transcriptomics generated in this study have been deposited in the Genome Sequence Archive (GSA) under accession code HRA002347 (https://ngdc.cncb.ac.cn/gsa-human/browse/HRA002347). The processed data of scRNA-seq and spatial transcriptomics generated in this study have been deposited in the Open Archive for Miscellaneous (OMIX) under accession code OMIX001122 (https://ngdc.cncb.ac.cn/omix/

release/OMIX001122). The proteomics data generated in this study have been deposited in the OMIX under accession code OMIX001127 (https://ngdc.cncb.ac.cn/omix/release/OMIX001127). All the above data belong to a same project in the National Genomics Data Center, China National Center for Bioinformation (CNCB-NGDC) under accession code of PRJCA009122 (https://ngdc.cncb.ac.cn/bioproject/browse/PRJCA009122). Source data are provided with this paper.

Field-specific reporting

Please select the one below that is the best fit for your research. If you are not sure, read the appropriate sections before making your selection.

☒ Life sciences ☐ Behavioural & social sciences ☐ Ecological, evolutionary & environmental sciences

For a reference copy of the document with all sections, see nature.com/documents/nr-reporting-summary-flat.pdf

Life sciences study design

All studies must disclose on these points even when the disclosure is negative.

Sample size	No statistical method was used to estimate sample size. For scRNA-seq, the sample size was based on published high quality scRNA-seq studies (see Referees: Ramachandran P, et al., Nature, 2019, 575, 512-518; Corridoni D, et al., Nat Med, 2020, 26, 1480-1490; MacParland SA, et al. Nat Commun, 2018, 9, 4383; Zhang M, et al. J Hepatol, 2020, 73, 1118-1130). According to the studies, we chosen 5 PBC samples and 4 control samples to perform scRNA-seq, which allowed us to perform statistical analysis on the obtained data. Other experiments were generally conducted with at least n = 3 biologically independent samples to guarantee reproducibility of the results. Sample size for each experiment is indicated in the figure legend.
Data exclusions	No data were excluded from analyses.
Replication	Experiments were conducted at least 3 times. Exact n-values are given in the figure legends.
Randomization	No randomization was used in the study due to patient data is observational.
Blinding	For all photomicrographs, the authors who did the experiments were blinded to group allocation during data collection and analysis

Reporting for specific materials, systems and methods

We require information from authors about some types of materials, experimental systems and methods used in many studies. Here, indicate whether each material, system or method listed is relevant to your study. If you are not sure if a list item applies to your research, read the appropriate section before selecting a response.

Materials & experimental systems

n/a	Involved in the study	
☐	☒	Antibodies
☒	☐	Eukaryotic cell lines
☒	☐	Palaeontology and archaeology
☐	☒	Animals and other organisms
☐	☒	Human research participants
☒	☐	Clinical data
☒	☐	Dual use research of concern

Methods

n/a	Involved in the study	
☒	☐	ChIP-seq
☐	☒	Flow cytometry
☒	☐	MRI-based neuroimaging

Antibodies

Antibodies used

All antibodies used in the study are listed in the Supplementary Table 14.

Antibodies for mouse immunofluorescence:

Ace2	Abcam, Cambridge, MA	ab108252
Duox2	Abcam, Cambridge, MA	ab97266
Ck19	Abcam, Cambridge, MA	ab52625
pIgR	Proteintech, Chicago, IL	22024-1-AP
Ck7	Abcam, Cambridge, MA	ab181598
Rabbit IgG	Proteintech, Chicago, IL	B900610
Mouse IgG	Proteintech, Chicago, IL	B900620

Antibodies for human immunofluorescence:

CD20	ZSGB-BIO, Beijing, BJ	ZA-0549

CD27	Proteintech, Chicago, IL	66308-1-Ig
IgD	ZSGB-BIO, Beijing, BJ	ZA-0443
CD138	ZSGB-BIO, Beijing, BJ	ZA-0584
ACE2	Proteintech, Chicago, IL	66699-1-lg
DUOX2	Abcam, Cambridge, MA	ab97266
CK19	Abcam, Cambridge, MA	ab52625
pIgR	Proteintech, Chicago, IL	22024-1-AP
CK7	Abcam, Cambridge, MA	ab181598
Rabbit IgG	Proteintech, Chicago, IL	B900610
Mouse IgG	Proteintech, Chicago, IL	B900620

Antibodies for mouse fluorescence activated cell sorting:

Duox2	Santa cruz, Dallas, TX	sc-398681
Ck19 Alexa Fluor® 647	Abcam, Cambridge, MA	ab192980
Ace2 CoraLite®488 Fluorescent Dye	Proteintech,Chicago, IL	CL488-66699
IgG2a heavy chain PE/Cy7®	Abcam, Cambridge, MA	ab130787
Isotype control to Duox2 PE/Cy7	BD Biosciences, Franklin Lakes,NJ	557872
Isotype control to Ck19 Alexa Fluor® 647	Abcam, Cambridge, MA	ab199093
Isotype control to Ace2 CoraLite®488	Proteintech,Chicago, IL	CL488-65124

Antibodies for human fluorescence activated cell sorting:

DUOX2	Santa cruz, Dallas, TX	sc-398681
CK19 Alexa Fluor® 647	Abcam, Cambridge, MA	ab192980
ACE2 CoraLite®488 Fluorescent Dye	Proteintech, Chicago, IL	CL488-66699
IgG2a heavy chain PE/Cy7®	Abcam, Cambridge, MA	ab130787
Isotype control to DUOX2 PE/Cy7	BD Biosciences, Franklin Lakes,NJ	557872
Isotype control to CK19 Alexa Fluor® 647	Abcam, Cambridge, MA	ab199093
Isotype control to ACE2 CoraLite®488	Proteintech,Chicago, IL	CL488-65124

Antibody for human immunohistochemistry:

CK7 Abcam, Cambridge, MA ab181598

Validation

All antibodies used in the study are commercially available with validation procedures described on the sites of the manufacture or relevant citation.
Ace2 for IF: https://www.abcam.cn/ace2-antibody-epr44352-ab108252.html
Duox2 for IF: https://www.abcam.cn/duox2-antibody-ab97266.html
Ck19 for IF: https://www.abcam.cn/cytokeratin-19-antibody-ep1580y-cytoskeleton-marker-ab52625.html
pIgR for IF: https://www.ptgcn.com/products/PIGR-Antibody-22024-1-AP.htm
Ck7/CK7 for IF and IHC: https://www.abcam.cn/cytokeratin-7-antibody-epr17078-cytoskeleton-marker-ab181598.html
Rabbit IgG for IF: https://www.ptgcn.com/products/Rabbit-IgG-B900610.htm
Mouse IgG for IF: https://www.ptgcn.com/products/Mouse-IgG-B900620.htm
CD20 for IF: https://www.sciencedirect.com/science/article/pii/S1198743X18302982
CD27 for IF: https://www.ptgcn.com/products/CD27-Antibody-66308-1-Ig.htm
IgD for IF: http://www.zsbio.com/product/ZA-0443
CD138 for IF: http://www.zsbio.com/product/ZA-0584
ACE2 for IF: https://www.ptgcn.com/products/ACE2-Antibody-66699-1-Ig.htm
DUOX2 for IF: https://www.scbt.com/p/duox2-antibody-e-8?requestFrom=search
CK19 for IF: https://www.abcam.cn/cytokeratin-19-antibody-ep1580y-cytoskeleton-marker-ab52625.html
Ck19/CK19 Alexa Fluor® 647 for FACS: https://www.abcam.cn/alexa-fluor-647-cytokeratin-19-antibody-ep1580y-ab192980.html
Ace2/ACE2 CoraLite®488 Fluorescent Dye for FACS: https://www.ptgcn.com/products/ACE2-Antibody-CL488-66699.htm
IgG2a heavy chain PE/Cy7® for FACS: https://www.abcam.cn/goat-mouse-igg2a-heavy-chain-pecy7-preadsorbed-ab130787.html
Isotype control to DUOX2 PE/Cy 7 for FACS: https://www.bdbiosciences.com/zh-cn/search-results?searchKey=557872
Isotype control to CK19 Alexa Fluor® 647 for FACS: https://www.abcam.cn/alexa-fluor-647-rabbit-igg-monoclonal-epr25a-isotype-control-ab199093.html
Isotype control to ACE2 CoraLite®488 for FACS: https://www.ptgcn.com/products/IgG1-Isotype-Control-Antibody-CL488-65124.htm

Animals and other organisms

Policy information about studies involving animals; ARRIVE guidelines recommended for reporting animal research

Laboratory animals

Wild type 7-week-old male C57BL/6 J mice were used for FACS and wild type 8- to 12-week-old male C57BL/6 J mice were used for isolating primary mouse hepatocytes.

Wild animals

The study did not involve wild animals.

Field-collected samples

The study did not involve samples collected from the field.

Ethics oversight	Animal protocols were approved by the Institutional Animal Care and Use Committee of the First Affiliated Hospital ofThird Military Medical University (AMUWEC20211648).

Note that full information on the approval of the study protocol must also be provided in the manuscript.

Human research participants

Policy information about studies involving human research participants

Population characteristics	For scRNA-seq and ST analyses, biopsied liver samples were obtained from 5 PBC patients with no history of ursodeoxycholic acid (UDCA) treatment (Supplementary Table 1). These PBC patients were diagnosed by an elevated serum alkaline phosphatase (ALP) levels and positive anti-mitochondrial antibodies (AMAs). Their liver tissues were biopsied to histologically exclude autoimmune hepatitis and other liver diseases (Supplementary Table 2 and Supplementary Fig. 1). Control surgical liver samples were obtained from 4 patients, who had no evidence of PBC, cholestasis, viral hepatitis, and autoimmune hepatitis (Supplementary Table 1). Three surgical liver samples were obtained from control patients for fluorescence-activated cell sorting (FACS) analysis of cholangiocytes (Supplementary Table 8). Moreover, surgical or biopsied liver samples from 16 control patients, 1 nonalcoholic steatohepatitis (NASH), 1 obstructive cholestasis (OC), 1 secondary sclerosing cholangitis (SSC) and 18 PBC patients, and an intestinal sample form 1 control patient were used for multiplex IF, RNAscope and liver histological assessments (Supplementary Table 9). The histological sections were evaluated by an experienced hepatopathologist in a blinded fashion and the degrees of fibrosis, bile duct loss, cholangitis and hepatitis activity in each sample were scored using the Ludwig and Nakanuma systems. Blood samples from 6 healthy volunteers and 10 PBC patients were used for proteomics (Supplementary Table 10). In addition, serum samples from 12 control subjects, 17 PBC patients, and 15 OC patients were used to quantify the levels of serum anti-pIgR antibodies by the ELISA assay (Supplementary Table 12).
Recruitment	Participants diagnosed at the First Affiliated Hospital of Third Military Medical University and the Xiangya Hospital of Central South University who meet our pre-defined criterion and are volunteer to participate in the study with a written informed consent were recruited. All participants are diagnosed according to international unified standards and have not received any treatment. There is no evidence for a self-selection bias.
Ethics oversight	The study protocol was approved by the Ethics Committees of the First Affiliated Hospital of Third Military Medical University and the Xiangya Hospital of Central South University. A corresponding written informed consent was obtained from individual subjects.

Note that full information on the approval of the study protocol must also be provided in the manuscript.

Flow Cytometry

Plots

Confirm that:

- [x] The axis labels state the marker and fluorochrome used (e.g. CD4-FITC).
- [x] The axis scales are clearly visible. Include numbers along axes only for bottom left plot of group (a 'group' is an analysis of identical markers).
- [x] All plots are contour plots with outliers or pseudocolor plots.
- [x] A numerical value for number of cells or percentage (with statistics) is provided.

Methodology

Sample preparation	The tissue pieces were digested in GEXSCOPE® Tissue Dissociation Solution (Singleron, Cat#1020012) at 37℃ for 20 minutes with consistent agitation and filtered through 70 µm sterile cell strainer (Falcon, Cat#352340), followed by centrifuged. After lysis of red blood cells, the remaining cells were examined for their viability (a cell preparation with >90% viability was used for subsequent experiments). A similar protocol was used for preparing mouse liver tissue single-cell suspension except for using 40% Percoll buffer (GE Healthcare, Cat#10283278) to wash the cell pellets once before red blood cell (RBC)lysis.
Instrument	BD FACSCanto II (BD Biosciences)
Software	FlowJo (version 10.6.2)
Cell population abundance	Cell population numbers can vary between each cell isolation and between the preparations from the different samples and species. However, this was irrelevant since the sorting was used to enrich for the target cell populations, marked with specific antibodies. The purity of the isolated cells was expressed as percentage of total cholangiocytes in post-sort samples
Gating strategy	Cells were firstly gated based on forward (FSC-A) and side (SSC-A) scatters to exclude debris. Dead cells were excluded based on their positive staining for DAPI. Next, a lineage marker, Ck19 was to distinguish cholangiocytes from liver cells. Cells from the positive fraction were further examined using anti-ACE2 and anti-DUOX2 antibodies.

- [x] Tick this box to confirm that a figure exemplifying the gating strategy is provided in the Supplementary Information.
